# Host microRNA analysis in cyprinid Herpesvirus-3 (CyHV-3) infected common carp

**DOI:** 10.1186/s12864-018-5266-9

**Published:** 2019-01-16

**Authors:** Michal Reichert, Anna Lukasik, Piotr Zielenkiewicz, Marek Matras, Joanna Maj-Paluch, Magdalena Stachnik, Ewa Borzym

**Affiliations:** 1 Department of Fish Diseases, National Veterinary Research Institute, 57 Partyzantow Avenue, 24-100 Pulawy, Poland; 2 Institute of Biochemistry and Biophysics, Polish Academy of Sciences, Pawinskiego 5a, 02-106 Warsaw, Poland; 3 Department of Plant Molecular Biology, Institute of Experimental Plant Biology and Biotechnology, University of Warsaw, 02-096 Warsaw, Poland

**Keywords:** Cellular miRNA expression, KHV, Herpesvirus, Common carp, Infection

## Abstract

**Background:**

The mechanism of latency and the ability of the cyprinid herpesvirus 3 (CyHV-3) to establish life-long infections in carp remains poorly understood. To explain the role of miRNAs in this process we applied a range of molecular tools including high-throughput sequencing of RNA libraries constructed from the blood samples of infected fish followed by bioinformatic and functional analyses which show that CyHV-3 profoundly influences the expression of host miRNAs in vivo.

**Results:**

We demonstrated the changed expression of 27 miRNAs in the clinical phase and 5 in the latent phase of infection. We also identified 23 novel, not previously reported sequences, from which 8 showed altered expressions in control phase, 10 in clinical phase and 5 in latent phase of infection.

**Conclusions:**

The results of our analysis expand the knowledge of common carp microRNAs engaged during CyHV-3 infection and provide a useful basis for the further study of the mechanism of CyHV-3 induced pathology.

**Electronic supplementary material:**

The online version of this article (10.1186/s12864-018-5266-9) contains supplementary material, which is available to authorized users.

## Background

Koi herpesvirus (KHV), also known as cyprinid herpesvirus 3 (CyHV-3) belongs to the genus *Cyprinivirus* of the family *Alloherpesviridae* comprising of herpesviruses infecting only fish and amphibians [1]. CyHV-3 has caused huge economic losses in common and koi carp culture industries worldwide since its emergence in the late 1990s [2, 3]. All known members of the *Herpesviridae* family demonstrate the ability to establish life-long infections in immunocompetent hosts. There are multiple known mechanisms of this immune evasion but we are still far from having a complete understanding of the underlying viral strategy. Among them, miRNAs driven gene expression regulation seems to be an important element of virus-host interplay enabling the creation of a beneficial environment for persistent virus infection. MicroRNAs (miRNAs) are a class of small non-coding RNAs (~ 22 nt) transcribed from the genomes of all multicellular eukaryotes and some viruses [4, 5]. Studies concerning the role of miRNA expression in virus infection has exploded in recent years. The common picture that has emerged from virus-host interaction is that virus encoded miRNAs are usually involved in this process and promote viral persistence through multiple mechanisms; evading the immune system, the inhibition of cell apoptosis and/or viral lytic cycle and the promotion of viral latency [5, 6]. In recent years an increasing body of evidence suggests that viruses exploit host miRNAs to control the process of infection. Some Herpesviruses are not exceptional in this context and are indeed able to exploit host miRNAs engaged in cellular pathways crucial for viral latency [7, 8]. It is interesting to note that only very limited data concerning the role of miRNAs during CyHV-3 infection of carp exists to date. We found only two reports [9, 10] about this subject, however, they focus mainly on the role of virus encoded miRNAs while leaving the role of the host components largely uninvestigated. This is not surprising as both studies were performed using an in vitro model of infection namely CCB (common carp brain) and KCF − 1 (caudal fin of koi) cell lines. Although analyses from one of the reports also indicate that cellular miRNAs are involved in the course of CyHV-3 infection, their value is greatly reduced due to the lack of a host immune component, which is a common shortcoming of in vitro models [10]. This shortcoming is especially significant in the herpesvirus infection as herpesviruses are masters in evading host immune response with miRNAs participating significantly in the underlying mechanisms. In order to gain knowledge concerning the role of host miRNAs in the KHV induced latency we focused our study on the analysis of host miRNA expression in CyHV-3 infected common carp. Several studies on the pathogenesis of CyHV-3 infection indicate that latency may be a distinctive feature of this representative of the family *Alloherpesviridae* which is similar to many members of the *Herpesviridae* family in this respect. Although numerous reports suggest that CyHV-3 may have existed in wild carp long before its recognition in the late 1990s, the detailed mechanism of its latency and the ability of the virus to establish life-long infections is far from being resolved [11–13]. To explore this intriguing biological phenomenon and bearing in mind the huge economic losses caused world-wide by KHV, we decided to carry out an experiment that would reproduce the natural infection of carp. To explain the role of miRNAs in this process we applied high-throughput sequencing of RNA libraries constructed from the blood samples of infected fish followed by bioinformatic and functional analyses which show that CyHV-3 profoundly influences the expression of host miRNAs in vivo.

## Methods

### Experimental fish

Forty-five healthy juvenile carp weighing 150 – 300 g, obtained from one of the Polish fish farms in kozienicki district of the mazowieckie voivodship with no history of KHV, were adapted to experimental conditions for one month. The fish were kept in a 400 L tank supplied with a constant flow of 90 L h^− 1^ of deep well water. Water temperature was maintained at 12 ± 1 °C, and a photoperiod 24:0 h light/dark regime was followed. Fish were fed to satiation twice a day. After adaptation fish were randomly divided into groups I and II (30 and 15 fish respectively). Then the fish from group I were exposed to the supernatant of a KHV-infected CCB cell culture harvested when the 90–95% cytopathic effect was evident. KHV of known genotype (U/I) used to infect group I was isolated from an outbreak of the disease at a common carp culturing farm and stored at −80^o^ C. Fish were exposed to infection through bath immersion for 45 min at a water temperature of 21 °C, the virus suspension had an infectivity of 3 × 10 ^2^ TCID_50_ ml^− 1^. Group II was mock infected with the cell culture medium and used as a control. After that, the fish were kept until 8 weeks after exposure. The progress of the infection was monitored using DNA samples prepared from fin sections of the fish and real-time PCR according to Gilad et al. [14]. Blood was collected 3 times from the fish of group II; one day before exposure, on the 7th day post infection when clinical signs become evident (lethargy, skin spots and skin haemorrhages, sunken eyes) and finally 8 weeks after exposure from the remaining 18 convalescent fish. Before blood collection the fish were anesthetized by immersion in 50 μg ml^− 1^ of tricaine methane sulfonate (MS-222). At the end of the experiment fish were euthanised by immersion into a 0.5 g L − 1 tricaine solution (Sigma-Aldrich). Blood samples were used for micro RNA isolation immediately after collection and then the RNA was kept at -80 °C until required. For library construction and NGS sequencing (Next Generation Sequencing), the best quality samples of blood RNA were chosen from the same three fish (s1, s2 and s3) in each phase of the infection (P1-control, P2-clinical, P3-latent). To this end, the concentration of RNA and the RIN (RNA integrity number) were determined using an Agilent 2100 bioanalyzer in conjunction with a RNA 6000 Pico LabChips kit. Description of the planned experiments was submitted to and approved by the Local Ethics Committee for Animal Experiments at the University of Life Sciences in Lublin. Decision no. 19/2013 issued on 12 March 2013.

### Small RNA library construction and high-throughput sequencing

MicroRNA was extracted from the carp whole blood using a dedicated miRNeasy Mini Kit (Qiagen) and purified according to the manufacturer’s instructions, eluated in 40 μl RNAse free water and stored at -80 °C until required.

Next generation sequencing (NGS) using an Illumina HiSeq 4000 sequencer was performed by Genomed SA (Poland) in partnership with BGI Tech Solutions (Hong Kong) according to the manufacturer’s protocol. Briefly, 1 μg of total RNA from each sample was used for library preparation with the NEBNext Multiplex Small RNA Library Prep Set for Illumina. RNA adapters were ligated to the 3′ and 5′ end of the RNA molecule and the adapter-ligated RNA was reverse transcribed into single-stranded cDNA. The cDNA was then PCR amplified using a common primer and a primer containing one of 9 index sequences. The introduction of the six-base indices at the PCR step allowed for the multiplexed sequencing of different samples in a single lane of the flowcell. One to 9 of these indexed cDNA libraries were pooled in equal amounts and gel purified together. The pooled library was hybridized to one lane of the eight-lane single-read flowcell on a cBot Cluster Generation System (Illumina) using a TruSeq Single-Read Cluster Kit (Illumina). The clustered flowcell was then loaded onto a HiSeq 4000 sequencer for a multiplexed sequencing run that consisted of a standard 36-cycle sequencing read with the addition of a six-cycle index read. A PhiX library was sequenced in lane 4 and used for calibration.

### sRNA data processing and mapping

Each of the 9 obtained, independent raw sRNA datasets was individually bioinformatically analyzed with a FASTX-toolkit (http://hannonlab.cshl.edu/fastx_toolkit/index.html) and cutadapt method to clean/remove: 1) adapter sequences, 2) reads shorter than 18 nt, 3) homo-polymer reads, 4) reads with N bases and 5) low quality reads, which had a quality score lower than 20. The remaining tags with identical sequences were collapsed. In the next step, the identification of miRNA molecules was performed. The BlastN method was used to find reads identical or similar to known miRNAs from common carp and *Danio rerio,* specifically reads which have: 1) no gaps in alignment with the reference, 2) no more than 1 mismatch in alignment with the reference, 3) sequences that differ in length by no more than 1 nucleotide from the reference alignment and 4) the E-value of the alignment ≤0.01. The unassigned reads were further used to identify a broader panel of different RNA types. This identification was completed in a hierarchical manner where the remaining reads from one step were used in the next one, specifically: miRNAs → pre-miRNAs → snoRNAs → rRNAs → tRNAs → snRNAs → mRNAs → mitochondrion genome (carp/*D. rerio*) → repeats → lncRNAs → pseudogenes → introns → carp genome → *D. rerio* genome → KHV genome → unassigned reads. In all of the listed steps, except the mapping onto certain genomes, the BlastN method was used with an E-value threshold of 0.01. In the pre-miRNAs, snoRNAs and snRNAs searches, no gaps and up to 1 mismatch were allowed in alignment with the reference, and the sequence coverage should not differ by more than 1 nucleotide. In the case of rRNAs, tRNAs and lncRNAs no gaps and up to 1 mismatch were allowed in alignment with the reference, and the sequence coverage should not differ by more than 2 nucleotides. In turn, for mRNAs, introns, pseudogenes and repeat associated sequences, no gaps and up to 2 mismatches were allowed in alignment with the reference, and the acceptable difference in sequence coverage was 2 nucleotides. The number of reference sequences used as well as databases for the RNA types mentioned above may be found in Additional file 1: Table S1. As mentioned above, in certain steps of the analysis unassigned reads were mapped onto: 1) the common carp mitochondrial genome (NC_001606.1, NCBI), 2) *D. rerio* mitochondrial genome (NC_002333.2, NCBI), 3) common carp genome (PRJEB7241, NCBI), 4) *D. rerio* genome (GRCz10, NCBI) and 5) KHV genome (NC_009127.1, NCBI). For this purpose the Bowtie2 approach [15] was used and set in such a way as to report only the best alignment where the entire read is mapped and no more than 1 mismatch is present. Since genome annotation is available only for *D. rerio* (GRCz10, NCBI), the properly mapped reads were annotated with the use of this data by the BEDTools intersect methods [16], with the indication that read mapping must be within range of only one annotation and both of them should be on the same strand. The results obtained at the blasting and mapping stages were parsed, and combined with the use of in-house python script to generate (separately for each RNA type) the read count matrix.

### miRNA differential expression and functional analysis

The miRNAs count matrices for each of 9 independent samples served as an input for differential expression analysis (paired samples mode). Two R packages were used for this purpose, namely DESeq2 [17] and edgeR [18]. The |log2 Fold Change| ≥ 1 and P-adjust value/FDR ≤ 0.05 were set as the threshold for significant differential expression. Results obtained by edgeR and DESeq2 were combined - only miRNAs designated by both of these methods were considered to be truly differentially expressed. The hierarchical clustering of the generated data (normalized and transformed by rlog) was implemented using the hclust function with the ward method, based on the Euclidean distance matrix. Before the described proper analysis, the Principle Component Analysis (PCA), as well as, samples clustering were performed with DESeq2 [17] which produced information about sample profile similarity and potential outliers.

To determine the potential function of differentially expressed carp miRNAs, the putative targets for the abovementioned molecules were predicted and annotated. At the time of analysis only 1844 *C. carpio* mRNA sequences were available (November 2016, NCBI). Therefore, to supplement this potential target set, the identification of coding sequences in carp was carried out. The 49,738 carp EST sequences (December 2016, NCBI) served as an input and were assembled with the use of the online EGassembler service [19] (custom parameters were set up). Subsequently, sequences coding polypeptides similar to 46,609 carp (December 2016, NCBI) or 46,451 *D. rerio* (December 2016, NCBI) proteins were selected using the BlastX method. A maximum of 1 gap and 2 mismatches in the alignment were allowed, as well as an alignment length difference not bigger than 2 nucleotides. The sequences collected in this manner, together with carp mRNAs, were used as potential targets in miRNA target prediction performed by the tools4miRs meta-server (www.tools4mirs.org) [20]. Four different prediction algorithms were incorporated into this analysis – PITA [21], miRmap [22], RNA22 [23] and miRanda [24]. For each approach restrictive parameters were set up, specifically: 1) PITA: -19 was the maximum ΔΔG score; 2) miRmap: no mismatches were allowed in the “seed” region (refers to 2–8 nt from 5′ end of the molecule), only 1 G:U wobble was allowed in the “seed” region, 6–7 was the “seed” length; 3) RNA22: only 1 G:U wobble was allowed in the “seed” region, no mismatches were allowed in the “seed” region, − 19 Kcal/mol was the minimal free energy of heteroduplex, specificity of 61%, sensitivity of 63%, 12 was the minimum number of paired-up bases in the heteroduplex, 7 was the “seed” length and 4) miRanda: 140 was the minimum score and − 19 Kcal/mol was the minimal free energy of the heteroduplex. Unique miRNA:target pairs, predicted by at least 3 out of the 4 algorithms used, were further considered. After this step, functional annotation was carried out. The Gene Ontology (GO) annotation (separately for up/down-regulated miRNAs from certain comparisons) was performed using the Blast2GO software (www.blast2go.com) in three main steps: 1) BlastX search against *D. rerio* proteins with an E-value threshold of 1e^− 10^, 2) Mapping Blast hits on GO terms and 3) Filtering annotations with an E-value threshold of 1e^− 10^. The GO enrichment analysis was also implemented with the use of Blast2GO software – the predicted targets for carp up/down-regulated miRNAs (from a certain comparison) served as a study set, in turn, all potential carp target sequences (protein coding) served as the population set. The Fisher’s Exact Test with the Benjamini-Hochberg correction for multiple testing and FDR threshold set at 0.05 were chosen for the calculations. The Kyoto Encyclopedia of Genes and Genomes (KEGG) pathway mapping was carried out on-line with the KAAS (KEGG Automatic Annotation Server) [25], which performed a BlastN comparison against *D. rerio* genes in the KEGG GENES database (custom parameters). The KEGG pathway enrichment analysis was performed using the clusterProfiler R package [26] - the predicted targets for carp up/down-regulated miRNAs (from a certain comparison) served as the study set, all potential carp target sequences (protein coding) served as the population set. The Hypergeometric Test with the Benjamini-Hochberg correction for multiple testing was used and the *P*-value/Q-value threshold was set at 0.05.

### Novel miRNAs prediction

The clean reads which were found to have a significant similarity to introns or remained unannotated after the “sRNA data processing and mapping” step, served as an input for novel miRNAs prediction. Novel carp miRNAs were suggested through the use of the mireap method (https://sourceforge.net/projects/mireap/, BGI) – defaulted parameters were set up for this analysis. Predicted precursors: 1) that were represented by reads counts ≥15; 2) has identified the miRNA* sequence and 3) in which the length of identified miRNA/miRNA* was not bigger than 24 nt were further considered. These precursors were validated by the HuntMi [27], miRBoost [28], and miRNAFold (online) [29] methods that classified them into real/pseudo pre-miRNAs. For each of these methods appropriate parameters were set up, specifically: 1) HuntMi – animal model was used; 2) miRBoost – cross-species model was used and delta was set at 0.25, and 3) miRNAFold – 150 was the window size, − 19 Kcal/mol was the energy threshold and the percentage of verified values was set at 80. The results obtained from the listed approaches were combined and only precursors which were confirmed by all 3 methods were considered to be pre-miRNAs. In the last step, the manual inspection of proposed precursors was performed to verify the presence of miRNA:miRNA* duplex overhangs, duplex pairing and its location on the hairpin. The novel carp pre-miRNAs were visualized using the UEA sRNA Workbench method [30].

### PCR validation of carp miRNAs during CyHV-3 infection

To confirm the results of NGS sequencing and described differential expression analysis 7 miRNAs (4 known and 3 novel) were selected for qRT-PCR analysis. MicroRNA was extracted from the carp blood as described earlier (chapter: Small RNA library construction and high-throughput sequencing). cDNA was synthesized from 10 ng miRNA using TaqMan MicroRNA Reverse Transcription Assay (Applied Biosystems). This assay uses looped-primer RT-PCR, a new real-time quantification method, to accurately detect mature miRNAs. Each 15- μl RT reaction consists of 100 mM dNTPs (with dTTP), 50 U/μl MultiScribe Reverse Transcriptase, 10x Reverse Transcription buffer, 20 U/ μl RNase Inhibitor, nuclease-free water, 3 μl RT-primer and 5 μl RNA sample. For miRNA we developed the following RT-primers: ccr-miR − 144 to detect a mature microRNA with sequence CUACAGUAUAGAUGAUGUACU; dre-miR-27a-5p to detect a mature microRNA with sequence AGGACUUAGCUCACUCUGUGAACA; miR-205-5p `with sequence UCCUUCAUUCCACCGGAGUCUG; miR − 1a-3p with sequence UGGAAUGUAAAGAAGUAUGUAU; F1-novel-miRNA4 with sequence GCTTGTTGTATGTGGGCCAGATA; F2-novel-miRNA9 with sequence ATTCACTCGCTCTCACGTCACTC; F3-novel-miRNA2 with sequence GCAAACCATCATGTGCTGCTCT. Cycling condition on the Bio-Rad Thermal Cycler were as follows: 16 °C for 30 min, then 42 °C for 30 min, 85 °C for 5 min and final temperature 4 °C. During the target amplification step, the Amplitaq Gold DNA polymerase amplifies target cDNA synthesized from the RNA sample, using sequence-specific primers from the TaqMan Assay Plates. TaqMan PCR Master Mix was mixed with product from the RT reaction. Each 20-μl PCR reaction consisted of 20x TaqMan MicroRNA Assay, TaqMan 2x Universal PCR master mix, nuclease free-water and RT-product. Cycling conditions on the Bio Rad CFX96 Touch Real-Time PCR Thermocycler were as follows: 95 °C for 10 min, followed by 40 cycles at 95 °C for 15 s and 60 °C for 60 s. All statistical analyses of the obtained results were carried out using Statistica (StatSoft) ver. 10 software.

## Results

### Experimental infection

All 30 fish from group I were successfully infected with KHV. Starting from the 7th day 12 fish became moribund and did not survive. The remaining 18 convalescent fish established a latent phase of infection and were kept until 8 weeks after exposure. The mechanism of latency in the case of CyHV-3 infection is not known but carp miRNAs identified in this study are also engaged in this mechanism as we demonstrated the changed expression of 27 miRNAs in the clinical phase (P2) and 5 in the latent phase (P3) of infection. We also identified 23 novel, not previously reported sequences, from which 8 showed altered expressions in control phase (P1), 10 in phase P2 and 5 in phaseP3.

### Analysis of small RNA tags and miRNA identification

The raw data collected from 9 sRNA libraries included around 10 million reads. Triplicates of these libraries (samples s1 (9), s2 (13) and s3 (14)) represent three phases of KHV infection, referred to here as phase P1, P2 and P3. After adapter trimming, tags quality cleaning and filtering (see Materials and Methods), around 9.4–9.9 million reads were obtained. The length of clean reads was analyzed and showed that the majority of sRNA sequences ranged from 21 to 23 nt in each library (Additional file 1: Figure S1). The peak was observed at 22 nt, which is consistent with the typical size of mature animal miRNAs [4]. Clean reads were further used to identify conserved miRNAs. Here, the restricted BlastN search against known carp and zebrafish miRNAs was performed and revealed that 259, 255 and 256 miRNA species are present in samples from infection phase P1, phase P2, and phase P3, respectively. The mean % of total clean reads representing miRNA molecules varies from 82% (P2) to 88% (P3) depending on the phase. The top 10 miRNAs species with the highest number of reads are as follows: 1) ccr-miR-21; 2) ccr-let-7a; 3) dre-let-7e; 4) ccr-miR-92a; 5) ccr-miR − 101a; 6) ccr-miR-26a; 7) ccr-miR-30d; 8) ccr-let-7 g; 9) ccr-miR − 128-3p and 10) dre-miR-462. Tags that remained unannotated after this processing were used for a series of rigorous blasting and mapping steps which revealed an abundance of different types of RNAs sequences, such as mRNAs, tRNAs, rRNAs, repeat sequences, snRNAs and others. Around 4% of total clean reads represent also part of the miRNAs precursors (a region outside the mature miRNA sequence). Since the common carp genome is available only in the preliminary (first) version without any annotation, the zebrafish RNA sequences, genome and its annotation were used additionally for this part of the analysis. Considering the KHV infection, it was interesting to evaluate the presence of sequences representing the genome of this virus. Only a few single reads, from only one phase (P2, clinical phase) were mapped to the KHV genome (Table 1). The number of these tags is however, not statistically significant. After the above-described reads annotation, only 0.2–0.4% of total clean reads were discarded from further consideration since they did not show any similarity to known common carp or zebrafish sequences. In turn, 4–7% of total clean reads remained unannotated. Detailed information regarding sRNA distribution among different RNA categories are summarized in Table 1 and Additional file 1: Figure S2.

**Table 1:** Annotation of total clean reads from samples (mean counts) representing each of three KHV infection phases

	Phase P1 (mean counts)	Phase P2 (mean counts)	Phase P3 (mean counts)
Raw reads	10,026,145	10,011,267	10,102,972
Clean reads	9,575,634	9,627,773	9,688,795
miRNA	8,308,461	7,858,105	8,474,366
pre-miRNA	400,781	425,229	420,086
tRNAs	124,225	223,161	100,263
rRNAs	83,870	135,885	60,105
mRNAs	148,946	233,832	136,264
repeats	11,664	9765	4616
snRNAs	5564	4722	3005
snoRNAs	22,981	18,904	15,896
lncRNA	3145	6176	3405
mitochondrion	1464	2224	2255
pseudogene	655	1092	580
Introns	42,078	34,257	37,516
Potential KHV sequence	0	4	0
Unannotated	398,478	645,125	408,956
Discarded	23,322	29,292	21,482

### Differentially expressed miRNAs

**Fig. 1:**
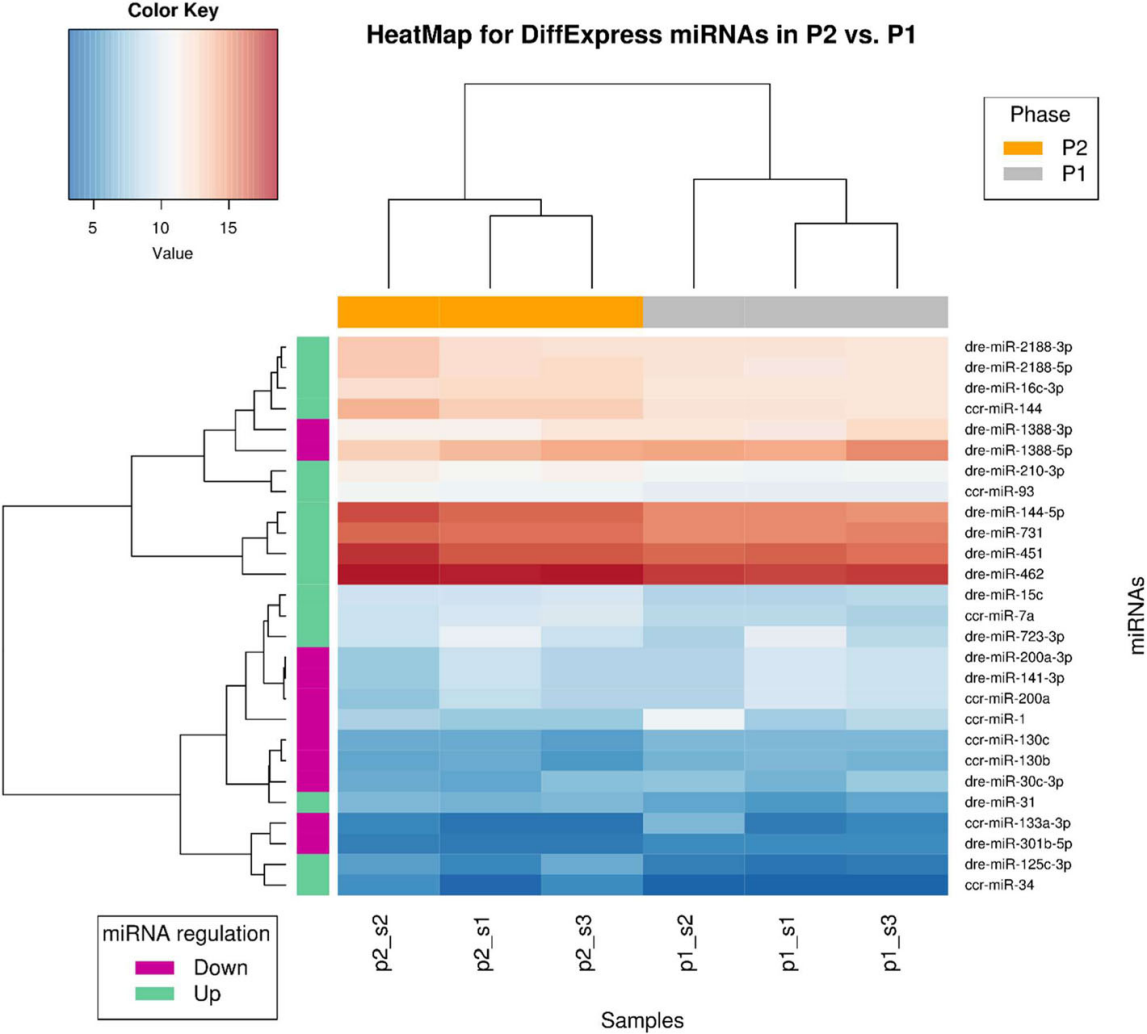
Heatmap of differentially expressed carp miRNAs - infection phase P2 vs. phase P1. Dendrograms represents results of hierarchical clustering by samples from analyzed phases (columns) and by differentially expressed miRNAs (rows). On heatmap, the darker the color the bigger (red) or smaller (blue) number of normalized reads counts that represent given carp miRNA molecule. On X axis, grey denotes samples from phase P1 and orange denotes samples from phase P2. On Y axis, green denotes up-regulated miRNAs and violet denotes down-regulated miRNAs. The |log2 Fold Change| ≥ 1 and P adjust value/FDR ≤ 0.05 were set as the threshold for significant differential expression

As a first step before appropriate differential expression analysis, the clustering heatmap was generated based on identified miRNA count matrices to evaluate miRNA profile similarities between samples/different phases and identify any potential outliers. The obtained heatmap, presented as Additional file 1: Figure S3, revealed that sample s1 from all 3 phases clustered together, which states that miRNA profiles from these samples are quite similar. Additionally, miRNA composition between phase P1 (control) and phase P3 (latent, without symptoms) also showed some similarity - samples s2 and s3 from the abovementioned phases clustered together (Additional file 1: Figure S3). The differential expression analysis (in paired sample mode) was implemented with the edgeR [18] and DESeq2 [17] methods – the consensus of their results was considered. Two comparisons were performed, namely: 1) the differentially expressed miRNAs between phase P2 and phase P1 (reference) named here as P2 vs. P1, and 2) the differentially expressed miRNAs between phase P3 and phase P1 (reference) named here as P3 vs. P1. In the P2 vs. P1 part of the analysis, 27 miRNAs were differentially expressed – including 16 up-regulated and 11 down-regulated molecules (Table 2 and Fig. 1). The significantly up-regulated miRNAs (with highest log2 Fold Change) include ccr-miR-34, dre-miR − 125c-3p, dre-miR − 144-5p and ccr-miR-7a (Table 2).

**Table 2:** Differentially expressed carp miRNAs in KHV infection phase P2 (clinical) as compared to phase P1 (control)

miRNA	Mean Reads Counts	log2FoldChange (Fold Change)	Regulation	P-value	Adjusted P-value
dre-miR-144-5p	83,682,03	2,29	UP	2,36E-19	5,21E-17
dre-miR-462	293,867,78	1,67	UP	1,35E-13	1,49E-11
ccr-miR-34	27,57	3,36	UP	7,80E-10	4,31E-08
ccr-miR-144	10,066,02	2,04	UP	4,59E-10	3,38E-08
dre-miR-125c-3p	47,93	2,60	UP	3,04E-08	1,12E-06
dre-miR-731	61,529,67	1,05	UP	4,94E-05	7,80E-04
dre-miR-451	125,823,57	1,29	UP	1,52E-04	1,53E-03
dre-miR-1388-5p	24,350,85	-1,45	DOWN	8,27E-05	1,08E-03
dre-miR-2188-5p	6211,25	1,40	UP	3,39E-07	1,07E-05
dre-miR-15c	489,63	1,67	UP	1,68E-09	7,44E-08
ccr-miR-7a	521,87	1,80	UP	7,11E-07	1,96E-05
ccr-miR-133a-3p	57,98	-1,90	DOWN	1,53E-04	1,53E-03
dre-miR-31	86,07	1,48	UP	1,97E-05	4,35E-04
dre-miR-301b-5p	28,44	-1,30	DOWN	4,42E-03	2,12E-02
dre-miR-1388-3p	3996,49	-1,05	DOWN	1,04E-05	2,55E-04
dre-miR-200a-3p	430,23	-1,22	DOWN	6,90E-05	1,02E-03
ccr-miR-200a	408,92	-1,23	DOWN	1,45E-04	1,53E-03
dre-miR-141-3p	421,76	-1,20	DOWN	1,67E-04	1,60E-03
ccr-miR-1	735,14	-1,86	DOWN	1,21E-04	1,48E-03
dre-miR-2188-3p	6166,01	1,05	UP	6,63E-04	4,89E-03
dre-miR-16c-3p	5547,82	1,02	UP	7,87E-05	1,08E-03
dre-miR-210-3p	2239,61	1,07	UP	2,30E-05	4,63E-04
ccr-miR-130b	81,94	-1,13	DOWN	8,37E-03	3,70E-02
ccr-miR-130c	89,33	-1,06	DOWN	3,65E-03	1,88E-02
ccr-miR-93	1344,69	1,04	UP	2,54E-05	4,68E-04
dre-miR-723-3p	767,64	1,10	UP	4,38E-03	2,12E-02
dre-miR-30c-3p	117,32	-1,03	DOWN	2,82E-03	1,52E-02

**Fig. 2:**
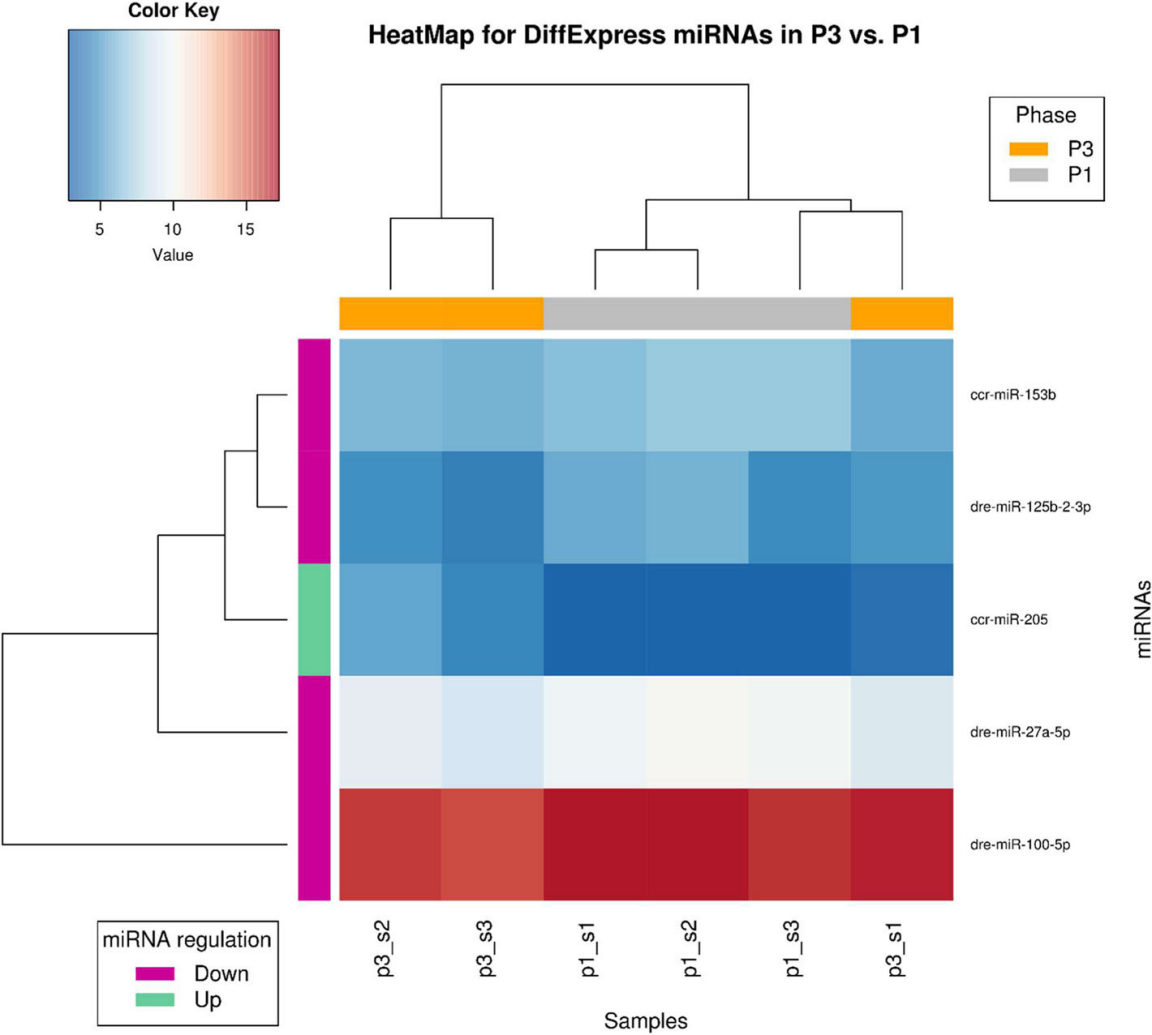
Heatmap of differentially expressed carp miRNAs - infection Phase P3 vs. phase P1. Dendrograms represents results of hierarchical clustering by samples from analyzed phases (columns) and by differentially expressed miRNAs (rows). On heatmap, the darker the color the bigger (red) or smaller (blue) number of normalized reads counts that represent given carp miRNA molecule. On X axis, grey denotes samples from phase P1 and orange denotes samples from phase P3. On Y axis, green denotes up-regulated miRNAs and violet denotes down-regulated miRNAs. The |log2 Fold Change| ≥ 1 and P-adjust value/FDR ≤ 0.05 were set as the threshold for significant differential expression

In turn, significantly down-regulated miRNAs (with lowest log2 Fold Change) are represented by ccr-miR − 133a-3p, ccr-miR − 1, dre-miR − 1388-5p and dre-miR-301b-5p (Table 2). The number of differentially expressed miRNAs between phase P3 and P1 (named as P3 vs. P1) is much smaller than that obtained from the previous comparison. Here, only 1 down-regulated miRNA, namely ccr-miR-205, and 4 up-regulated miRNAs were obtained (Table 3 and Fig. 2). The latter comprise ccr-miR-153b, dre-miR-27a-5p, dre-miR-100-5p and dre-miR125b-2-3p (Table 3).

**Table 3:** Differentially expressed carp miRNAs in KHV infection phase P3 (latent, without symptoms) as compared to phase P1 (control)

miRNA	Mean Reads Counts	log2FoldChange (Fold Change)	Regulation	P-value	Adjusted P-value
ccr-miR-205	24,98	2,45	UP	4,26E-08	5,41E-06
ccr-miR-153b	85,31	-1,29	DOWN	9,94E-06	0,000361
dre-miR-27a-5p	794,96	-1,39	DOWN	2,20E-05	0,000699
dre-miR-100-5p	113,102,59	-1,04	DOWN	7,28E-05	0,002056
dre-miR-125b-2-3p	41,57	-1,35	DOWN	0,000209	0,004271

### miRNA target prediction and functional annotation

In order to better understand the role of differentially expressed, conserved miRNAs in carp (especially during KHV infection), the prediction and annotation of their targets were performed. The 10,600 common carp EST sequences encoding proteins, together with 1782 carp mRNAs sequences served as potential targets sets for 3 appropriate groups of miRNAs, specifically: 1) all differentially expressed miRNAs from the P3 vs. P1 comparison (together up-regulated and down-regulated); 2) down-regulated miRNAs from the P2 vs. P1 comparison and 3) up-regulated miRNAs from the P2 vs. P1 comparison. The target prediction was performed with the tools4miRs service and incorporated 4 different approaches. Targets for miRNAs were considered for further analysis only if they were predicted by a minimum of 3 methods. In this manner the number of unique targets obtained for the abovementioned miRNA groups was as follows: 1) 44; 2) 449 and 3) 2107. The detailed list of predicted miRNA: target pairs may be found in the Additional file 2: Tables S2-S4.

Subsequently, the GO terms annotation and KEGG pathway mapping for predicted targets were performed to identify functions and pathways that are actively regulated by differentially expressed miRNAs (separately for miRNA groups 1–3). This was implemented with the use of the Blast2GO and KAAS approach [25], respectively. Since currently, GO annotations and KEGG reference maps for common carp genes are missing, the only solution to perform such an analysis is to use *Danio rerio* annotations/reference pathways. The obtained results have revealed that down-regulated miRNAs from the P2 vs. P1 phase comparison are potentially involved in inter alia: the immune system process, response to stress, signal transduction, cell development, MAPK signaling pathway (ko04010), Herpes simplex infection pathway (ko05168), Epstein-Barr virus infection (ko05169), NOD-like receptor signaling pathway (ko04621), biosynthesis of secondary metabolites (ko01110) and Th17 cell differentiation (ko04659). Analogous annotations were obtained for up-regulated miRNAs from the P2 vs. P1 phase comparison, the number of mapped sequences is however, expectedly different. Additionally, the abovementioned miRNAs may also regulate: apoptosis (ko04210), endocytosis (ko04144) and ribosome (ko03010). In turn, differentially expressed miRNAs from the P3 vs. P1 phase comparison could play important roles in, e.g., regulation of apoptotic process, programmed cell death, the positive regulation of the immune system process, receptor binding and the PI3K-Akt signaling pathway (ko04151). The pie chart graphs presenting GO terms distribution, the full list of GO terms annotation and mapped KEGG pathways may be found as Additional file 1: Figures S4-S6, Additional file 3: Tables S5-S7 and Additional file 4: Tables S8-S10, respectively.

**Fig. 3:**
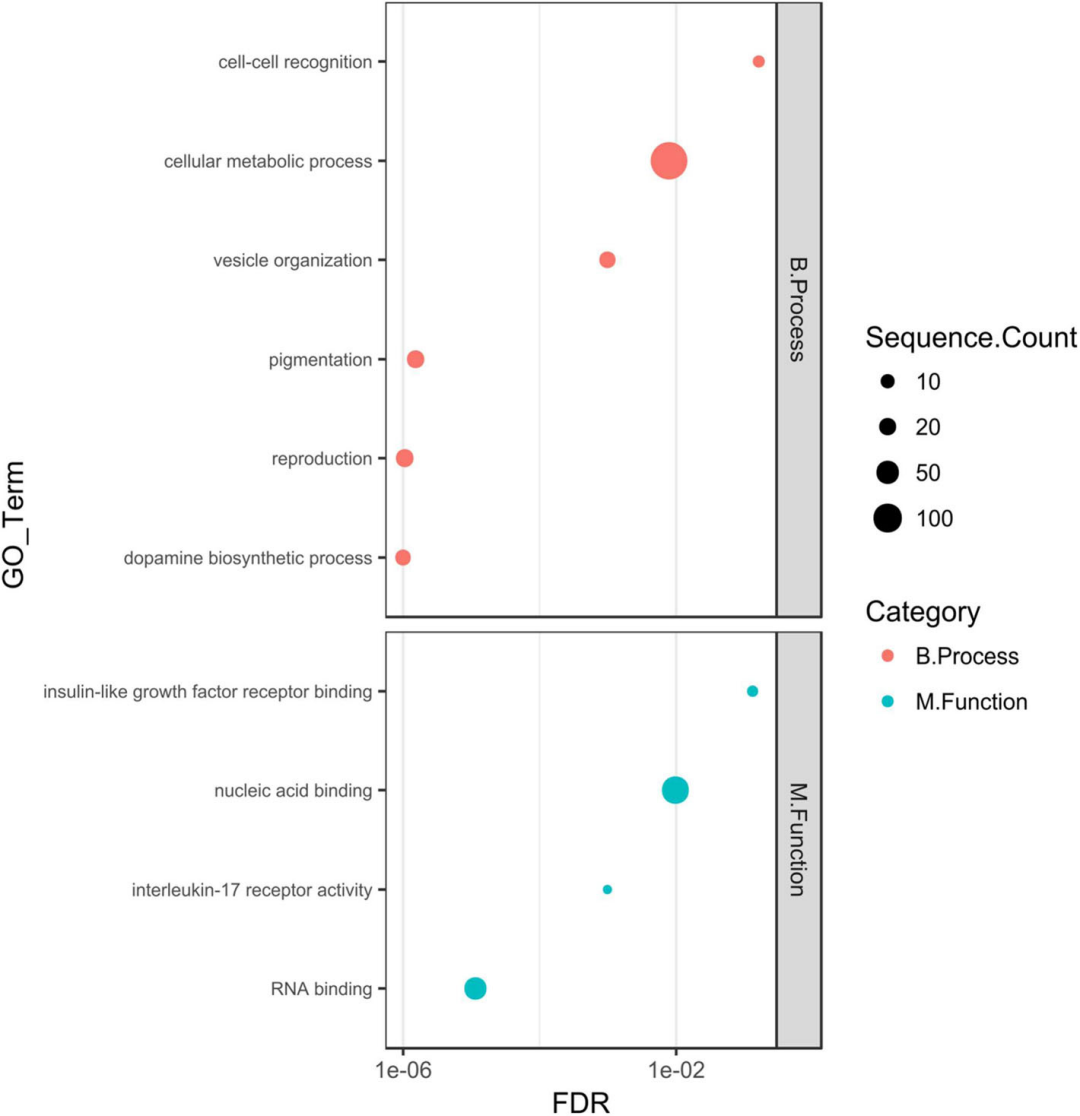
Selected over-represented GO terms for targets of carp down regulated miRNAs (P2 vs. P1 phase comparison). The GO terms enrichment analysis was performed by the Blast2GO software. The carp targets predicted for down-regulated miRNAs were used as a test set. All carp protein coding sequences were used as background. Figure represent selected enriched GO terms from the “Biological Process” and “Molecular Function” categories. The size of dot denotes number of targets annotated by given GO term. The FDR ≤ 0.05 was set as the threshold for significant GO term enrichment

**Fig. 4:**
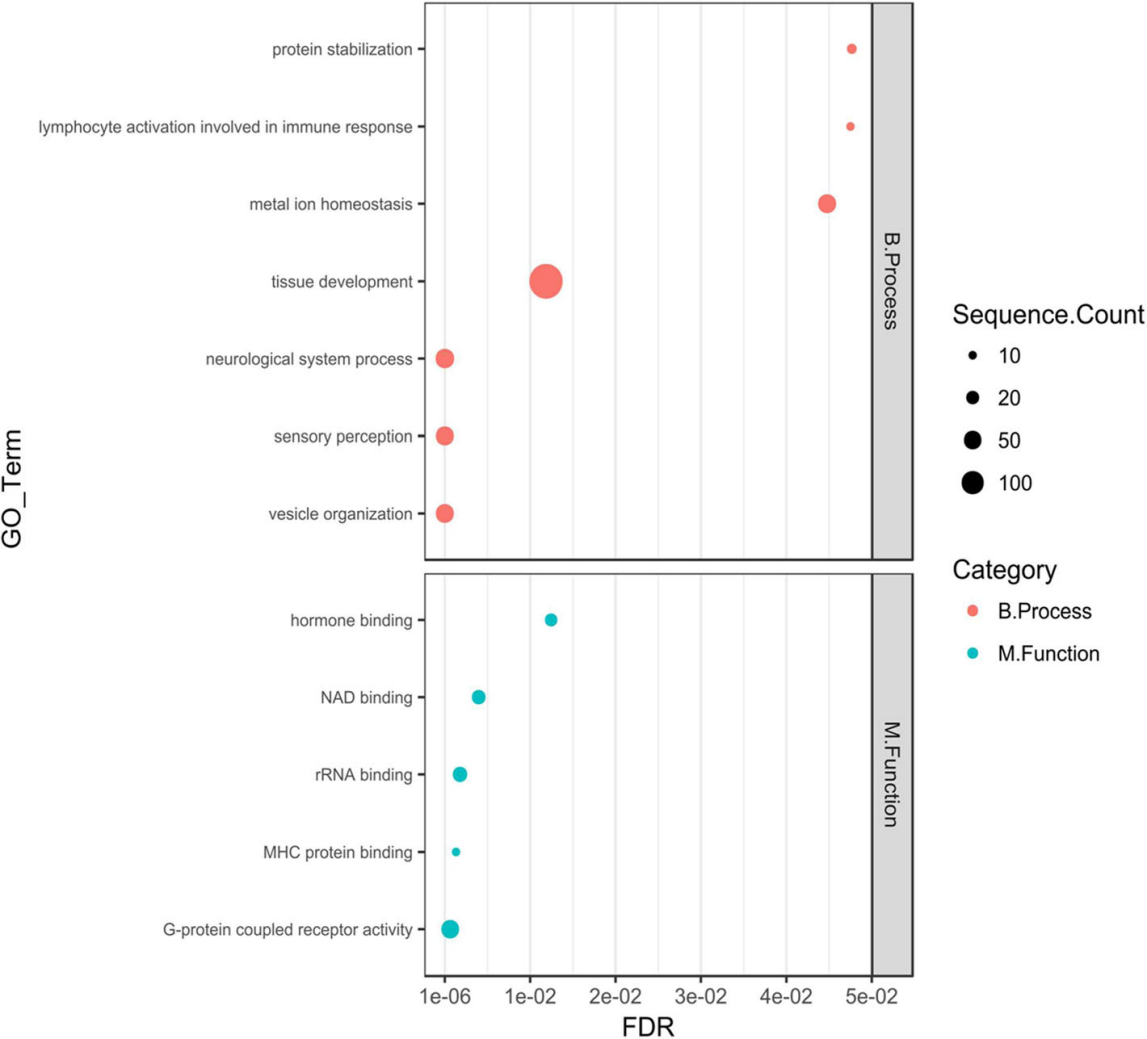
Selected over-represented GO terms for targets of carp up regulated miRNAs (P2 vs. P1 phase comparison). The GO terms enrichment analysis was performed by the Blast2GO software. The carp targets predicted for up-regulated miRNAs were used as a test set. All carp protein coding sequences were used as background. Figure represent selected enriched GO terms from the “Biological Process” and “Molecular Function” categories. The size of dot denotes number of targets annotated by given GO term. The FDR ≤ 0.05 was set as the threshold for significant GO term enrichment

To statistically supplement the above-described functional annotations, the GO terms and KEGG pathway enrichment analysis were carried out (separately for miRNA groups 1–3), using Blast2GO and clusterProfiler [26] approaches, respectively. Several over-represented GO terms were obtained for only two groups of differentially expressed miRNAs, namely for up-regulated and down-regulated miRNAs from the P2 vs. P1 phase comparisons. This part of the functional annotation has shown that targets for the abovementioned down-regulated molecules could play essential roles in a broad range of biological processes or have diverse molecular functions, such as, vesicle organization, pigmentation, reproduction, cell-cell recognition, dopamine biosynthetic process, RNA binding, insulin-like growth factor receptor binding and interleukin-17 receptor activity (Fig. 3). In the case of up-regulated miRNA molecules, their targets are potentially involved in inter alia*:* protein stabilization, lymphocytes activation involved in immune response, metal ion homeostasis, sensory perception, neurological system processes, G-protein coupled receptor signaling pathway, tissue development, hormone binding, NAD binding and MHC protein binding (Fig. 4). A full list of over-represented GO terms is presented in Additional file 5: Tables S11-S12 and Additional file 1: Figures S5-S6. The KEGG enrichment analysis revealed only two significantly over-represented pathways, namely “Fatty acid metabolism” (dre01212; adjusted *P*-value 0.048) for up-regulated miRNAs from the P2 vs. P1 phase comparison and “Necroptosis” (dre04217; adjusted P-value 0.001) for down-regulated miRNAs from the P2 vs. P1 phase comparison (Additional file 5: Tables S13-S14). The abovementioned KEGG pathways with highlighted mapped target genes are presented as Additional file 1: Figure S7 and S8.

### Novel *C. carpio* miRNA molecules

Tags which remained unannotated after the reads processing step or showed significant similarity to intron sequences were used in the mireap method to predict novel pre-miRNAs. Proposed hairpin precursors were further evaluated using 3 different approaches that classified them into real and pseudo pre-miRNAs. To improve the quality of predicted molecules, the selected stem-loop structures were evaluated manually. As a result of the described steps, 8 novel miRNAs from phase P1, 10 novel miRNAs from phase P2 and 5 novel miRNAs from phase P3 were obtained. The total number of clean reads representing predicted miRNAs ranged from 14 to 3020. In turn, the Minimum Folding Energy (MFE) of the proposed precursors was between − 25.1 and − 47.6 Kcal/mol. Detailed information regarding these novel molecules may be found in the Additional file 6: Tables S15-S17. The predicted hairpin structures of novel miRNAs are shown in the Additional file 1: Figure S9-S11.

### PCR validation of carp miRNAs during CyHV-3 infection

We confirmed the expression of identified carp miRNAs through the use of quantitative real-time RT- PCR using previously selected 7 miRNAs (4 known and 3 novel ones). Among the four known miRNAs: 1) two differentially expressed in phase P2 versus phase P1 of CyHV-3 infection -namely, one up-regulated (miR-144) and one down-regulated (mir-1) and 2) two miRNAs, differentially expressed in phase P3 versus phase P1 - namely one up-regulated (miR-205) and one down-regulated (miR-27a) were selected. Also the three novel miRNAs; miRNA4 (phase P1), miRNA9 (phase P2) and miRNA2 (phase P3) were chosen. RNA samples collected from 18 convalescent carps in each phase of infection were used for real-time RT- PCR evaluation. Among them were also samples from 3 carps (s1, s2, s3) used for library construction. Validation analysis confirmed the significant up-regulation of mir-144 and mir-205 in phase P2 and phase P3 respectively as compared to phase P1. Also, significant down-regulation of mir-1 and mir-27a-5p in phase P2 and phase P3 respectively as compared to phase P1 was confirmed (Table 4). The results of qRT-PCR supported our decision not to expand validation to the remaining miRNAs.

**Table 4:** qRT-PCR validation of carp miRNAs expression during CyHV-3 infection

miRNA	Fold change (P2 v. P1) of miRNA expression.	Ct mean value $\overline{X}$ ± SD,*N* = 18 measured by qRT-PCR, (P2 v. P1)	Student’s t-test and *p* value	Fold change (P3 v. P1) of miRNA expression.	Ct mean value $\overline{X}$ ± SD,N = 18 measured by qRT-PCR, (P3 v. P1)	Student’s t-test and p value
mir-1	-1,86/DOWN	P2: 29,92 ± 3,93 P1: 27,96 ± 4,64	*t* = 2,69 *p* = 0,02*		P3: 29,72 ± 3,41 P1: 27,96 ± 4,64	*t* = 1,96 p = 0,07
mir-205		P2: 30,76 ± 2,84 P1: 32,57 ± 2,67	*t* = −1,71 p = 0,11	2,45/UP	P3: 30,07 ± 2,82 P1: 32,57 ± 2,67	*t* = −2,49 p = 0,03*
miR 144	2,04/UP	P2: 23,81 ± 1,54 P1: 25,11 ± 1,41	*t* = −3,42 *p* = 0,004*		P3: 24,19 ± 1,86 P1: 25,11 ± 1,41	t = −2,14 *p* = 0,05
miR-27a-5p		P2: 34,93 ± 0,99 P1: 35,23 ± 1,16	t = −1,25 p = 0,23	-1,39/DOWN	P3: 35,97 ± 1,13 P1: 35,23 ± 1,16	t = 2,48 p = 0,03*

Fold change – average change of miRNAs expression in 3 carps (s1, s2, s3) from each phase of infection), Ct value – comparison of average Ct value of qRT-PCR of 18 samples from experimental fish (including s1, s2, s3) between phases. Student’s t-test values for mean Ct values between phases(*) statistically significant differences between mean Ct values for *p-*value < 0.05

## Discussion

The profiling of microRNAs has been until now performed in just a few species of the family *Cyprinidae* i.e. bighead carp (*Hypophthalmichthys nobilis*) [31], silver carp (*Hypophthalmichthys molitrix*) [32], *Grass carp* (*Ctenopharyngodon idella*) [33], *Crucian carp (Carassius auratus* [27] and Tibetan naked carp *Gymnocypris przewalskii* [34]. The most economically significant representative of the family i.e. common carp was the subject of such investigation focused mainly on the identification and understanding of the role of microRNAs in physiological processes [35–37] but also in different pathological conditions like microcystin-LR exposure [32] or *Flavobacterium columnare* infection [38]. Very little is known concerning the role of microRNA expression during CyHV3 infection. We found only two reports [9, 10] about this subject, however, they focus mainly on the virus encoded miRNAs while leaving the role of the host components largely uninvestigated. The authors of one of these reports [9] furthermore confirmed the expression of two identified virus encoded miRNAs in vivo i.e. in the gills of infected carp. Both studies were performed using an in vitro model of infection, namely CCB and KCF-1 cell lines. Although for one of these reports [10] cellular miRNAs were also analysed in the course of CyHV-3 infection their value is greatly reduced because of the lack of a host immune component, which is a common shortcoming of the in vitro models [10]. To overcome this obstacle we performed an experiment in vivo using CyHV3 infected carps. In this case bioinformatic and functional analyses allowed us to compare miRNA expression at different phases of infection and to functionally annotate differentially expressed molecules. The functional relevance of the changed expression of identified miRNAs in viral latency and pathogenesis is then discussed.

In our study we identified in total 32 miRNAs differentially expressed in the course of CyHV-3 infection as compare to 19 common carp miRNAs found upregulated in CyHV-3 infected KCF-1 cell lines. Analysis showed that all identified miRNAs are different from those identified in the CyHV-3 infected cell line. This may confirm the profound differences in the engagement of miRNAs in living fish versus cell culture. However, we studied their expression at two different time points depending on the clinical status of the infection i.e. at the clinical and latent phases (P2 and P3 respectively). We found 27 miRNAs differentially expressed in the clinical and 5 miRNAs in the latent phase. The small number of differentially expressed miRNAs in the latent versus clinical phase reflects the probable recovery of the host to more physiological (healthy) conditions with parallel physiological expression of relevant miRNAs. Such a situation is appropriate to the in vivo conditions therefore the results of the cited cell culture study [10] have only a limited value in the study of the pathogenesis of CyHV-3 infection in common carp and cannot be compared. Validation of four highly dysregulated miRNAs and three novel miRNAs confirmed the good correlation between findings using both NGS and qRT-PCR. miRNAs up-regulated in phase P2 or P3 comparing with phase P1 showed the same pattern using the qRT-PCR method. The same was true in the case of down-regulated miRNAs (Table 4). What’s more, the expression patterns of selected miRNAs demonstrated in three fish were similar in a cohort of 18 fish. This proves that the results of our study are repeatable and reliable.

Bioinformatics analysis revealed that samples from one fish (no. 1) clustered together regardless of whether it was phase P1, P2 or P3. It may be helpful to explain that the clinical symptoms in the case of fish no.1 were very limited. Therefore one may assume that fish quickly reached a latency phase and good clinical status. This is not surprising as the measurement of the virus load in blood cells of fish no.1 showed the lowest counts as compared with the two other fish (no. 2 and 3). Eight weeks after exposure we could not detect the virus in blood cells of fish no. 1. Absence or a very low virus replication rate in fish no. 1 may result in a lack of changes in miRNA expression in this case.

Our study revealed only subtle changes in the expression of miRNAs between phase P1 and P3 which was evidenced i.e. by clustering most of the samples from these phases together. We found only 5 miRNAs that were differentially expressed in phase P3 as compared with 27 miRNAs in phase P2. This result probably reflects the quick reversion to homeostasis as the unique feature of latency in CyHV-3 infected common carp. Lin at al. [39] revealed that virus replication in the WBCs of latently infected carps increased 3–4 times during CyHV-3 reactivation and then dropped to the basic level. It is reasonable to assume that virus-host co-existence may reach a kind of equilibrium that allows the persistent presence of the virus in host cells without the induction of clinical symptoms. The 5 detected miRNAs may be the part of the machinery controlling this equilibrium.

The study of miRNA targets and their functional annotation has revealed several pathways and a broad range of biological processes in which miRNAs are involved in the course of CyHV-3 infection (see Results section). Among them, KEGG enrichment analysis revealed only two significantly over-represented pathways, namely “Fatty acid metabolism” for up-regulated and “Necroptosis” for down-regulated miRNAs from P2 vs. P1 phase comparison.

Only single reads (1–9) from phase P2 samples were mapped to the Koi Herpesvirus genome; The identified single reads are not significant but this is interesting when we take into account that they were found only in phase P2 (clinical phase). This may suggest that replication of CyHV-3 in phase P3 (latent phase) was absent or highly restricted as compared to the phase P2.

The identification of the fatty acid metabolism pathway as being engaged in the course of CyHV-3 infection is not surprising as fatty acid biosynthesis is essential for the replication of many viruses. Similarly, Munger et al. [40] showed that fatty acid biosynthesis is essential for the replication of HCMV (*Human cytomegalovirus*), which is closely related to the CyHV-3 virus. They found that HCMV infection upregulates much of the central carbon metabolic flux, as well as efflux to nucleotide and fatty acid biosynthesis [40]. miRNAs may participate in this regulation through a mechanism similar to the one described by Lin et al. [41] as they found changes in the expression of miR-125b and miR-205 in the case of zebrafish (*Danio rerio*) exposed to triclosan (known fatty acid synthase inhibitor). Lin et al. also demonstrated that miR-125b and miR-205 were associated with fatty acid synthesis pathways and that regulating mechanisms involve the PI3K-Akt signaling pathway. We also found a changed expression of miR-125b and miR-205 and the engagement of the PI3K-Akt signaling pathway in convalescent carp (phase P3 of infection). The involvement of miR-125b and miR-205 in the regulation of transcription factors connected with fatty acid metabolism pathway may prove that despite the subclinical course, viral replication still takes place in phase P3 of infection although probably at a very low level.

Another significantly over-represented pathway targeted by down-regulated miRNAs was necroptosis, which, like apoptosis is a form of programmed cell death used by the host to eliminate infected cells before the production of progeny virions. Viruses tend to evade cell-death based elimination and evolve strategies to manipulate cell-death signaling pathways. CyHV-3 is not exceptional in this matter therefore the engagement of the necroptosis pathway during the course of infection is not entirely unexpected. Morphologically, necroptosis is characterized by membrane disruption and organelle swelling. Recently, necroptosis has been considered as a regulated form of necrosis activated in the absence of caspase 8 activity [42]. Our study revealed that differentially expressed miRNAs are engaged both in the regulation of apoptosis as well as necroptosis. However, a significantly over-represented necroptosis pathway was found only for the P2 vs. P1phase comparison while apoptosis engagement was evidenced both for P2 vs. P1 as well as for the P3 vs P1 phases comparison. Such results may imply that necroptosis is a host cell preferred mechanism of antiviral defense in the acute, clinical phase of CyHV-3 infection where viral replication reaches the highest rate. In turn, apoptosis is more common in the latent phase (phase P3) characterized by a minimal rate of virus replication.

Recent investigations show that necroptosis can also act as an alternative cell death pathway in cases of viral infections where the virus produces proteins capable of blocking apoptosis signaling pathways [43]. Viruses, and in particular herpesviruses can modulate cell death pathways and determine which one will be executed; apoptosis or necrosis [44, 45]. The central role played by the cytosolic complex called ripoptosome consisted of the receptor interacting with protein kinase RIP in the form of RIP1-RIP3 heterodimer, Fas-associated protein (in the case of TNF family death receptor signaling), Casp8 and the long form of FLICE inhibitory protein (FLIP_L_). RIP activates mixed lineage kinase domain-like (MLKL) protein and, when Casp8 activity is inhibited, triggers oligomerization that leads to an amyloid-like complex that recruits MLKL into a necrosome that localizes to membranes and directs the final steps in necroptosis leading to membrane leakage [45]. Human herpesviruses (HSV1 and HSV2) adopted strategy enables them to overcome both cell-autonomous death pathways. Virus encoded proteins ICP6 and ICP10 suppress death receptor-dependent apoptosis by the interaction of the large subunit of ribonucleotide reductase with the death effector domains of Casp8 but they also encode RHIM signaling competitors acting as viral suppressors of RIP3- dependent necroptosis (reviewed in 11).

According to KEGG pathway mapping of predicted targets (for up and down-regulated miRNAs from P1 vs. P2 phase comparison) the miRNAs identified in our study are also components of the Herpes simplex virus (28 targets for up-regulated and 11 for down-regulated miRNAs) and the Epstein-Barr virus (EBV) (35 targets for up-regulated and 13 for down-regulated miRNAs) infection pathways (Additional file 4: Tables S8-S10). Therefore, it is reasonable to assume that the mechanisms of latency of CyHV-3 and human herpesviruses (HSV and EBV) can exploit similar pathways in infected cells, which is not particularly striking as all three viruses are taxonomically related (Order *Herpesvirales*). HSV and EBV are however much more learned herpesviruses and detailed pathways and crucial virus encoded molecules interfering with host cell pathways are known. In the case of CyHV-3 this is still a great challenge. Special attention should also be paid to the PI3K-Akt signaling pathway (ko04151) and MAPK signaling pathway (ko04010) as both are activated in the course of HSV and EBV infections [46, 47]. All aspects of their activation and the exact role they play are still the subject of intensive studies however they are vital for virus propagation [48–50]. In particular, MAPK/ERK kinases (extracellular signal-regulated kinase) participate in EBV virus production and reactivation from latency [49], interleukin- 6 and interleukin-8 production [51] and ER stress-mediated apoptosis [52]. The involvement of the MAPK signaling pathway is also exploited by HSV as part of the immune evasion mechanism. The key element is the direct inhibition of TCR signal transduction in HSV infected cells by dephosphorylating LAT (linker for the activation of T cells) - an adapter molecule that anchors multiple signaling complexes [52]. As a result, downstream events in the TCR cascade, such as calcium mobilization and the activation of MAPK in the Ras pathway are stopped and CTL (cytotoxic T lymphocytes) cytolytic and cytokine effector functions become blocked [53]. Another mechanism is the inhibition of the TAP-mediated loading of peptides (transporter associated with antigen processing) on MHC class I (major histocompatibility complex) by HSV encoded ICP47 protein which leads to the down-regulation of MHC in HSV-infected cells and to decreased CTL killing in vitro [54]. We have previously reported MHC I down-regulation in carp cells infected with CyHV-3 as an important immune evasion mechanism [55]. However, the details of this mechanism are not known. Given that among the predicted miRNAs targets, numerous components linked to HSV infection (28 for up-regulated and 13 for down-regulated miRNAs targets in the P2 vs. P1 phase comparison) were identified (Additional file 4: Tables S8-S10), it would not be surprising that CyHV-3 has evolved similar immune evasion mechanisms like in the case of HSV infections. Our analysis may therefore consist of the basis for furthermore detailed study aiming to search for crucial, CyHV-3 encoded molecules manipulating the host cellular machinery.

Over-represented GO terms related to MHC protein binding were also obtained for up-regulated miRNA targets (P2 vs. P1 phase comparison) (Fig. 4). This is a very interesting finding as it might help to explain the mechanism of CyHV-3 induced latency in virus infected fish. One of the key elements of this latency i.e. immune evasion strategy is common for the most studied human herpesviruses (e.g. HSV, EBV, HCMV or KSHV - Kaposi’s sarcoma-associated herpesvirus) immune evasion strategy [56, 57]. Such a strategy in the case of representatives of the *Alloherpesviridae* family is an almost completely unexplored field of research. The only one report suggesting the important inhibitory role of CyHV-3 in MHC class I expression does not explain in detail the underlying mechanism [55]. Therefore the identification of molecules/pathways involved in MHC regulation will help to dissect this important mechanism of CyHV-3 immune evasion.

The identification of numerous targets for CyHV-3 induced host miRNAs known to participate in the Herpes Simplex virus pathway suggests that CyHV-3 may share at least some molecules or cellular pathways exploited by HSV. For such a conclusion to be fully justified requires of course the functional validation of the miRNAs targets using available techniques e.g. CLASH, RIP-CHIP, PAR-CLIP etc., however that was not the subject of this study. Nevertheless, the first evidence of the similarity between signaling pathways involved in CyHV-3 and human herpesvirus (HSV) infection may also prove that the mechanisms of latency share some common elements and that latency may be an evolutionary conserved mechanism [58]. The detailed dissection of carp miRNAs targetome involved in the course of CyHV-3 infection and their functional relevance in viral latency and pathogenesis requires further study using different approaches and technologies.

The proposal of novel miRNAs (8 in phase P1, 10 in phase P2 and 5 in phase P3) illustrates that knowledge concerning the role of host miRNAs in the case of CyHV-3 infection is still in its infancy and offers an excellent experimental model to study virus-host miRNAs interaction using computational techniques.

## Conclusions

This is to the best of our knowledge the first report about host miRNA expression in the course of CyHV-3 infection of fish as previous reports [9, 10] involved experiments using CyHV-3 infected cell cultures. Therefore the results are more reliable and reflect biological processes in naturally infected carp with the host immune system as a crucial component of the host-virus interplay. What’s more, the results presented have expanded the knowledge base concerning common carp microRNAs and provided a useful basis for the further study of the mechanism of CyHV-3 induced pathology.

## Additional files

Additional file 1: **Figure S1-S11**. All supplementary figures. **Table S1.** Common carp and D. rerio reference sequences used for sRNA data processing - BlastN search. (PDF 4341 kb)

Additional file 2: **Table S2-S4.** Results of the target prediction for up-regulated, down-regulated miRNAs from P2 vs. P1 comparison and down-regulated and up-regulated miRNAs from P3 vs. P1 comparison. (XLS 460 kb)

Additional file 3: **Table S5-S7.** Results of the GO term annotation for targets of down-regulated, up regulated, miRNAs from P2 vs. P1 comparison and up-regulated and dwon-regulated miRNAs from P3 vs. P1 comparison. (XLS 2150 kb)

Additional file 4: **Table S8-S10.** Results of the KEGG pathway mapping analysis for down-regulated, up-regulated, miRNA targets from P2 vs. P1 comparison and up-regulated and down-regulated miRNA targets from P3 vs. P1 comparison. (XLS 90 kb)

Additional file 5: **Table S11-S14.** List of over-represented GO terms for targets of down-regulated, up-regulated carp miRNAs. List of enriched KEGG pathways for targets of down-regulated, up-regulated carp miRNAs (P2 vs. P1 phase comparison). (XLS 410 kb)

Additional file 6: **Table S15-S17.** Comrehensive list of novel carp miRNAs identified in samples representing infection phase P1, P2, P3. (XLS 30 kb)

## Abbreviations

CCB: Cell line from common carp brain
CTL: Cytotoxic T lymphocytes
CyHV-3: Cyprinid herpesvirus 3
EBV: Epstein-Barr virus
ERK: Extracellular signal-regulated kinase
FLIP_L_-FLICE: Long form of FLICE inhibitory protein (FLIPL)
GO: Gene Ontology
HCMV: Human cytomegalovirus
HSV: Human herpesvirus
KAAS: KEGG Automatic Annotation Server
KCF − 1: Cell line from caudal fin of koi
KEGG: Kyoto Encyclopedie of Genes and Genomes
KHV: Koi herpesvirus
KSHV: Kaposi’s sarcoma-associated herpesvirus
LAT: Linker for the activation of T cells
MAPK: Mitogen-activated protein kinase
MFE: Minimum Folding Energy
MHC: Major histocompatibility complex
miRNAs: MicroRNAs - a class of small non-coding RNAs
MLKL: Mixed lineage kinase domain-like protein
NGS: Next-Generation sequencing
PI3K: Phosphatidylinositide 3-kinases
RIP3: Receptor-interacting protein
TAP: Transporter associated with antigen processing
TCR: T-cell receptor
