# Supplementary material for: Host microRNA analysis in cyprinid Herpesvirus-3 (CyHV-3) infected common carp
Source: BMC Genomics. 2019 Jan 16;20:46. doi: 10.1186/s12864-018-5266-9 (PMC6337785; doi:10.1186/s12864-018-5266-9)

miRNA analysis in Common carp infected with KHV – SUPPLEMENTARY MATERIAL

Supplementary Table S1. Common carp and *D. rerio* reference sequences used for sRNA data processing - BlastN search.

RNA Type	Number of Unique Reference Sequences	Reference Database
miRNA	420	miRBase v.21
pre-miRNA	480	miRBase v.21
snoRNA	244	Silva v.122; NCBI (November 2016); Rfam v.12.1
rRNA	4777	Silva v.122; NCBI (November 2016); Rfam v.12.1
tRNA	8135	Silva v.122; NCBI (November 2016); Rfam v.12.1
snRNA	392	Silva v.122; NCBI (November 2016); Rfam v.12.1
mRNA	68324	RefSeq (protein-coding, November 2016)
Repeat associated sequences	2313	Repbase (November 2016)
lncRNA	48	Silva v.122; NCBI (November 2016); Rfam v.12.1
Pseudogenes	104	NCBI (November 2016)
Introns	40375	UCSC (November 2016)

Supplementary Figure S1. Length distribution of clean sRNA reads from samples representing KHV infection phase P1 (A), phase P2 (B) and phase P3 (C).

A)

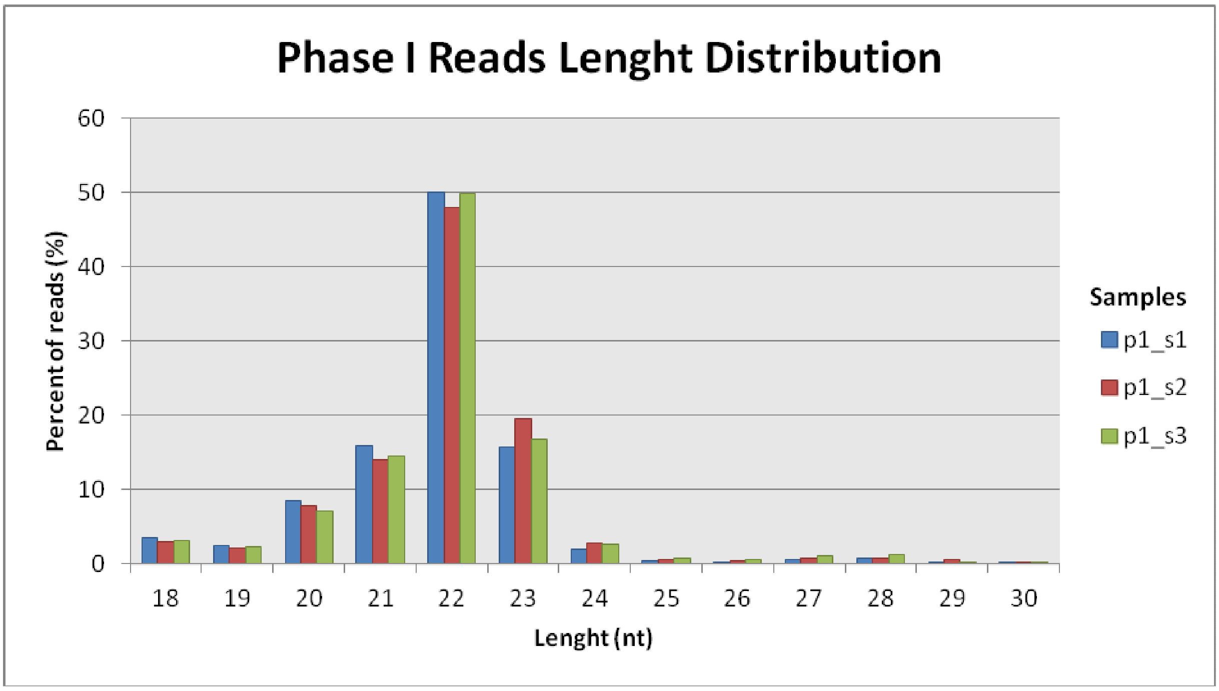

Phase I Reads Lenght Distribution
Percent of reads (%)
60
50
40
30
20
10
0
18
19
20
21
22
23
24
25
26
27
28
29
30
Lenght (nt)
Samples
p1_s1
p1_s2
p1_s3

B)

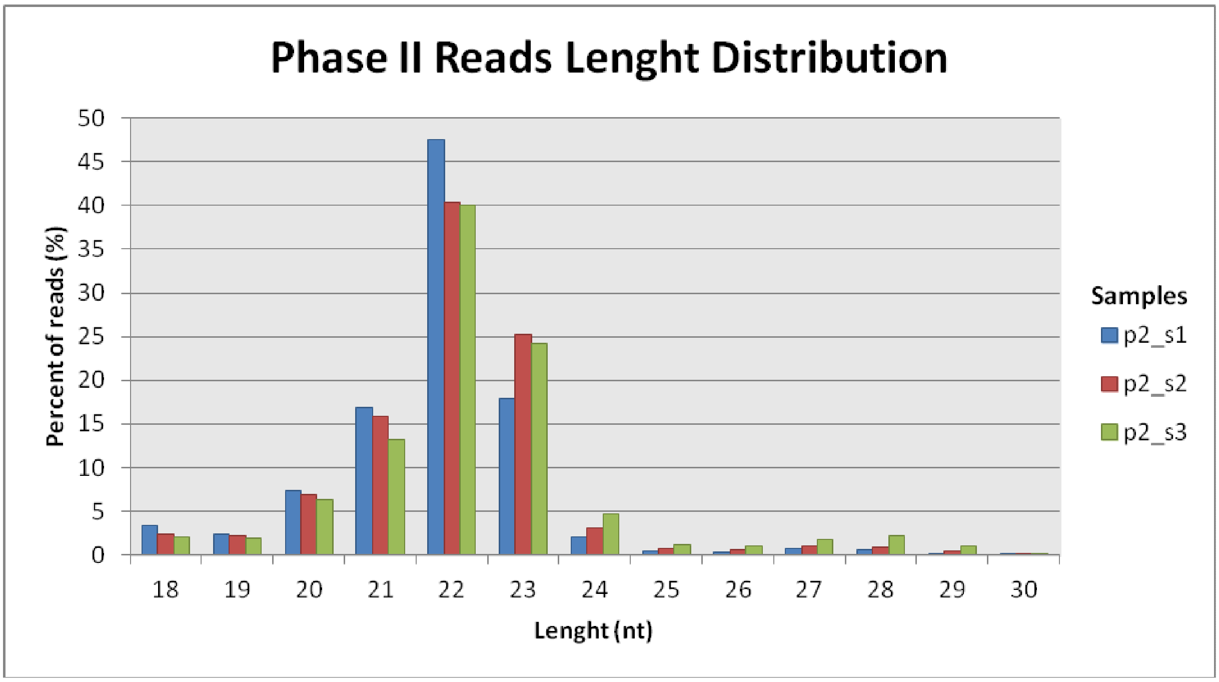

Phase II Reads Lenght Distribution
Percent of reads (%)
50
45
40
35
30
25
20
15
10
5
0
18
19
20
21
22
23
24
25
26
27
28
29
30
Lenght (nt)
Samples
p2_s1
p2_s2
p2_s3

C)

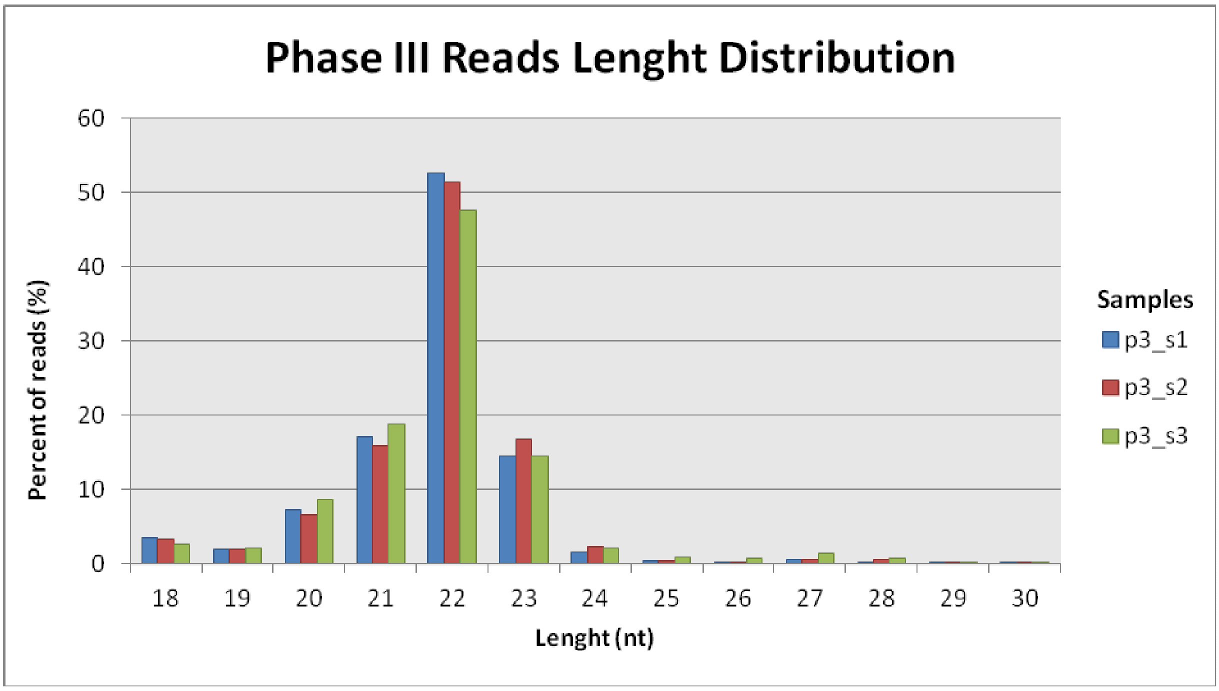

Supplementary Figure S2. Distribution of total clean reads from samples (mean counts) representing 3 KHV infection phases among different types of RNA.

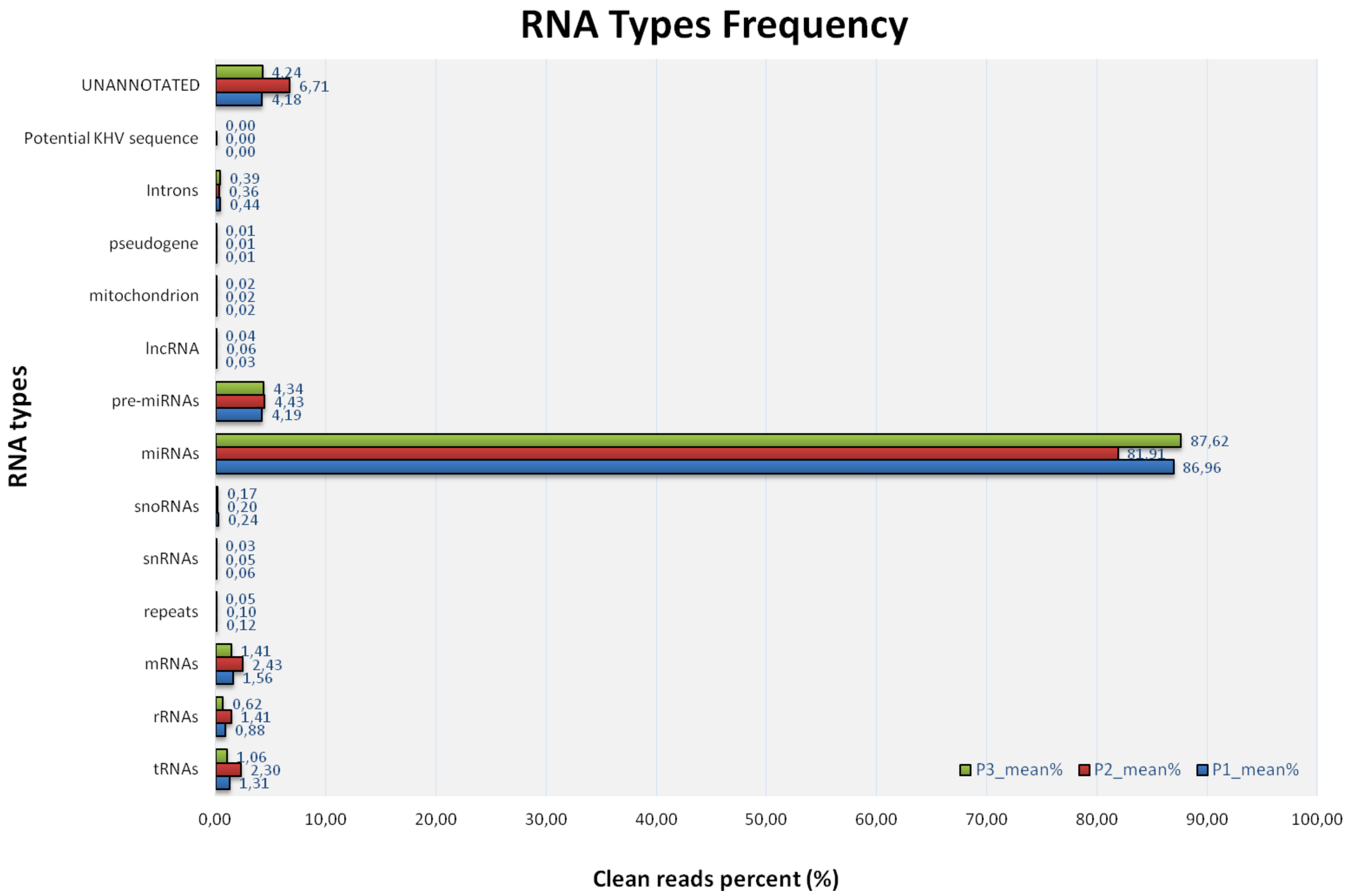
RNA Types Frequency
UNANNOTATED
4,24
6,71
4,18
Potential KHV sequence
0,00
0,00
0,00
Introns
0,39
0,36
0,44
pseudogene
0,01
0,01
0,01
mitochondrion
0,02
0,02
0,02
lncRNA
0,04
0,06
0,03
pre-miRNAs
4,34
4,43
4,19
miRNAs
87,62
81,91
86,96
snoRNAs
0,17
0,20
0,24
snRNAs
0,03
0,05
0,06
repeats
0,05
0,10
0,12
mRNAs
1,41
2,43
1,56
rRNAs
0,62
1,41
0,88
tRNAs
1,06
2,30
1,31
P3_mean%
P2_mean%
P1_mean%
RNA types
0,00
10,00
20,00
30,00
40,00
50,00
60,00
70,00
80,00
90,00
100,00
Clean reads percent (%)

Supplementary Figure S3. Heatmap of sample-to-sample distance (similarity). The heatmap was build using DESeq2 on normalized (rlog) miRNA reads counts from all 9 samples (all 3 infection phases). The darker the color the smaller distance and bigger similarity between samples.

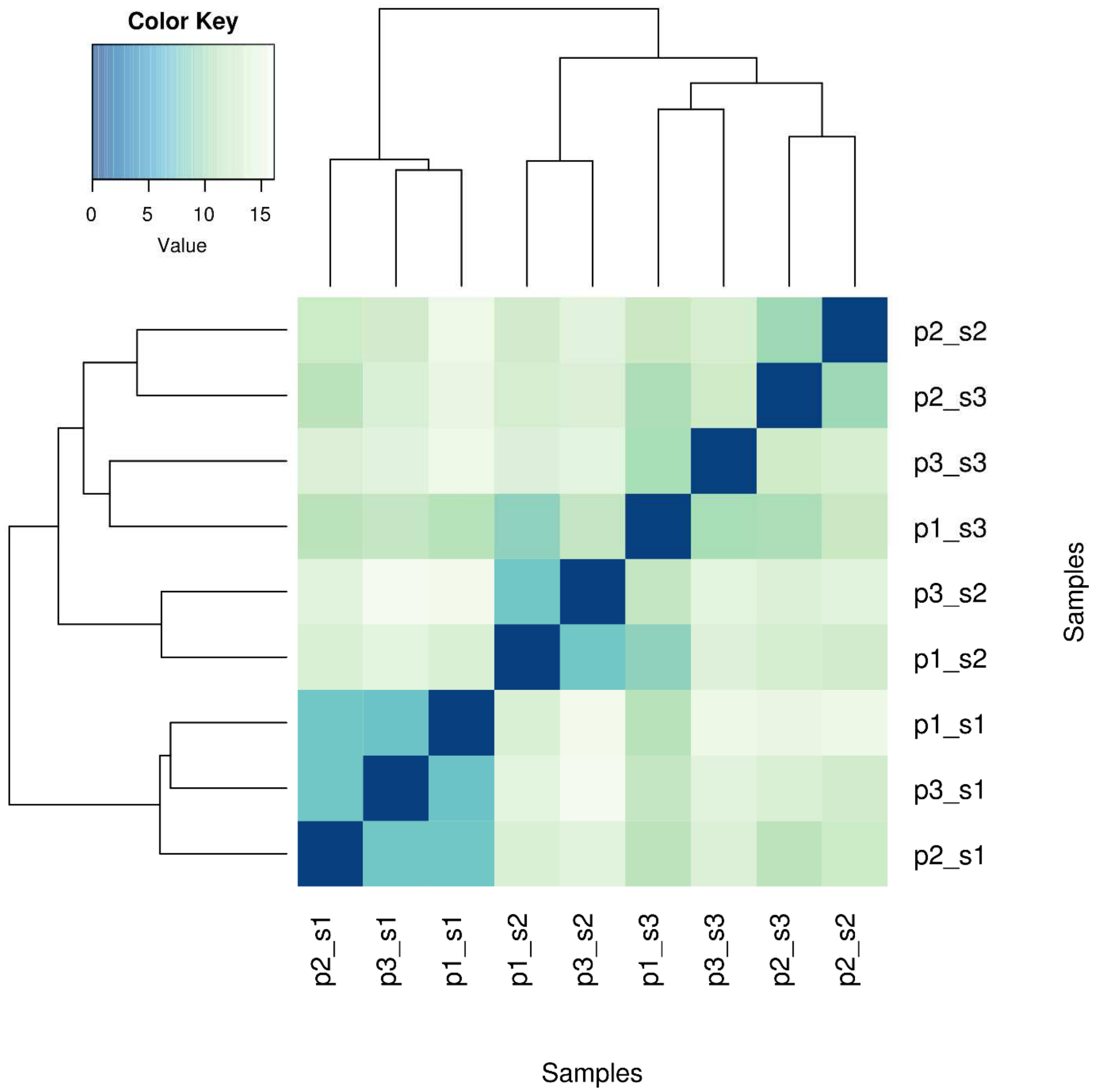

Supplementary Figure S4. Results of the GO term annotation for targets of up-regulated miRNAs from P2 vs. P1 comparison. The GO terms annotation was performed by the Blast2GO software. Figure represents the pie chart graph of GO terms distribution from "Biological Process" (A), "Molecular Function" (B) and "Cellular Component" (C) category – numbers in brackets represent number of annotated carp sequences.

A)

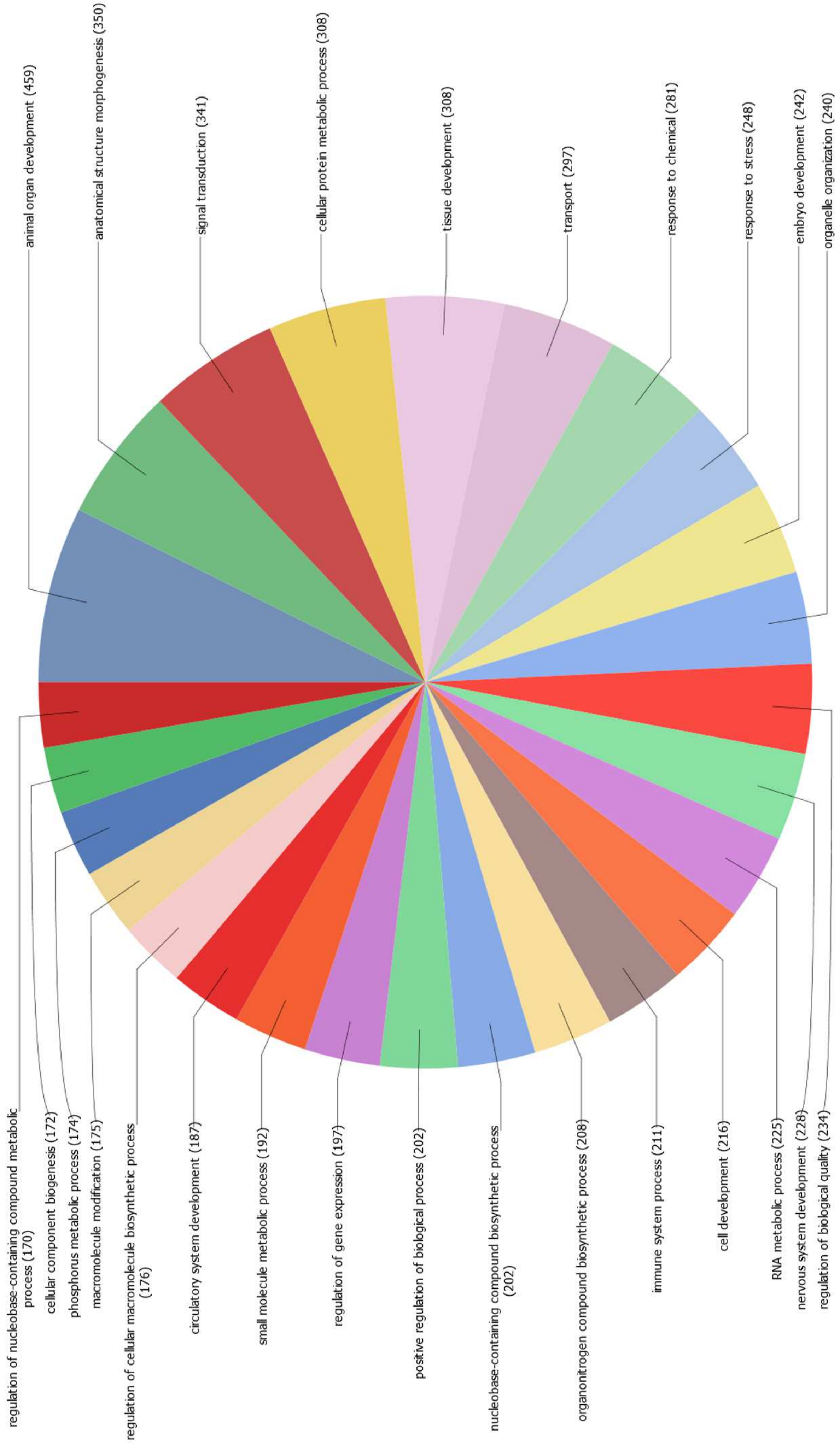

animal organ development (459)
anatomical structure morphogenesis (350)
signal transduction (341)
cellular protein metabolic process (308)
tissue development (308)
transport (297)
response to chemical (281)
response to stress (248)
embryo development (242)
organelle organization (240)
regulation of biological quality (234)
nervous system development (228)
RNA metabolic process (225)
cell development (216)
immune system process (211)
organonitrogen compound biosynthetic process (208)
nucleobase-containing compound biosynthetic process (202)
positive regulation of biological process (202)
regulation of gene expression (197)
small molecule metabolic process (192)
circulatory system development (187)
regulation of cellular macromolecule biosynthetic process (176)
macromolecule modification (175)
phosphorus metabolic process (174)
cellular component biogenesis (172)
regulation of nucleobase-containing compound metabolic process (170)

B)

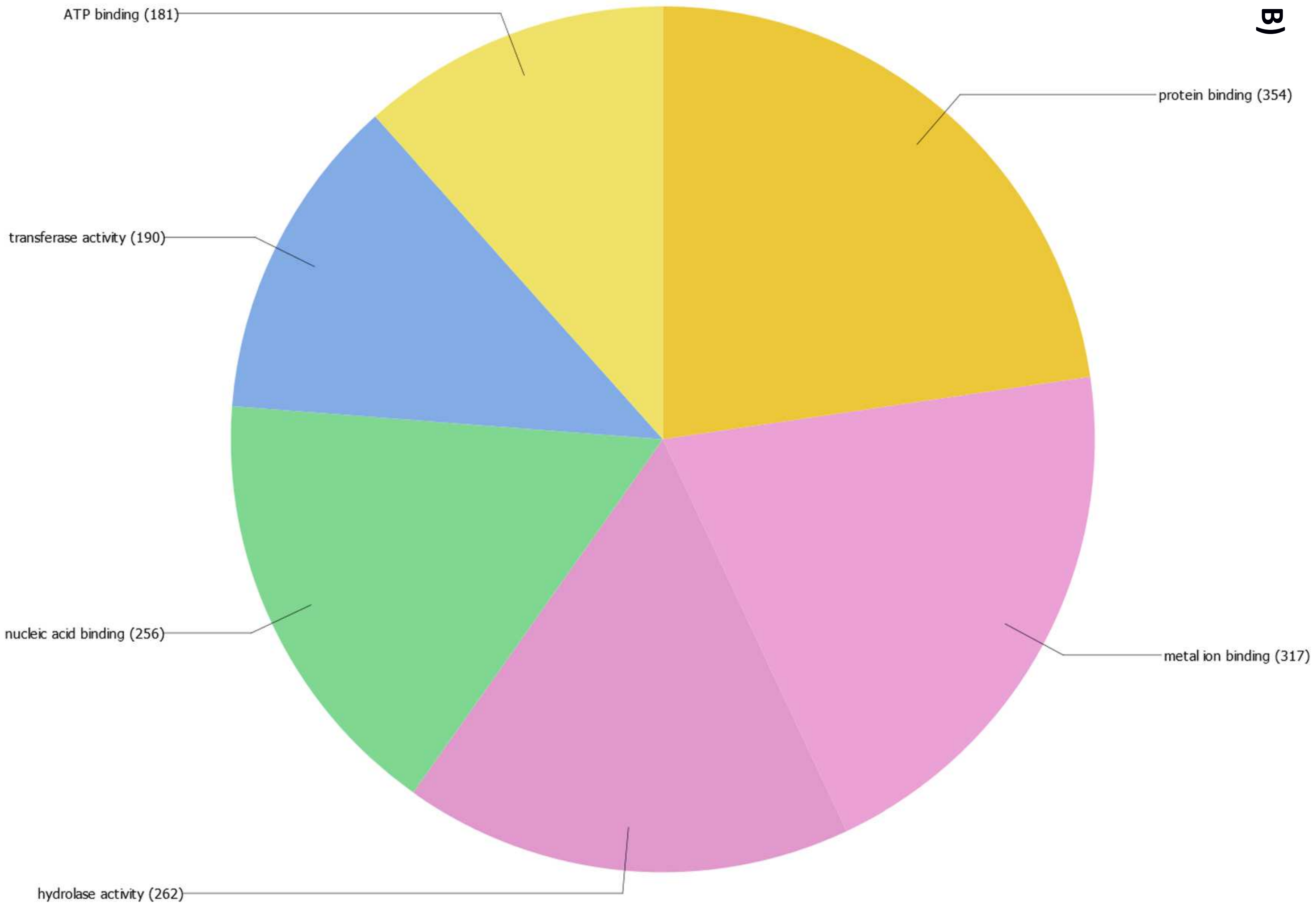

ATP binding (181)
protein binding (354)
transferase activity (190)
nucleic acid binding (256)
metal ion binding (317)
hydrolase activity (262)

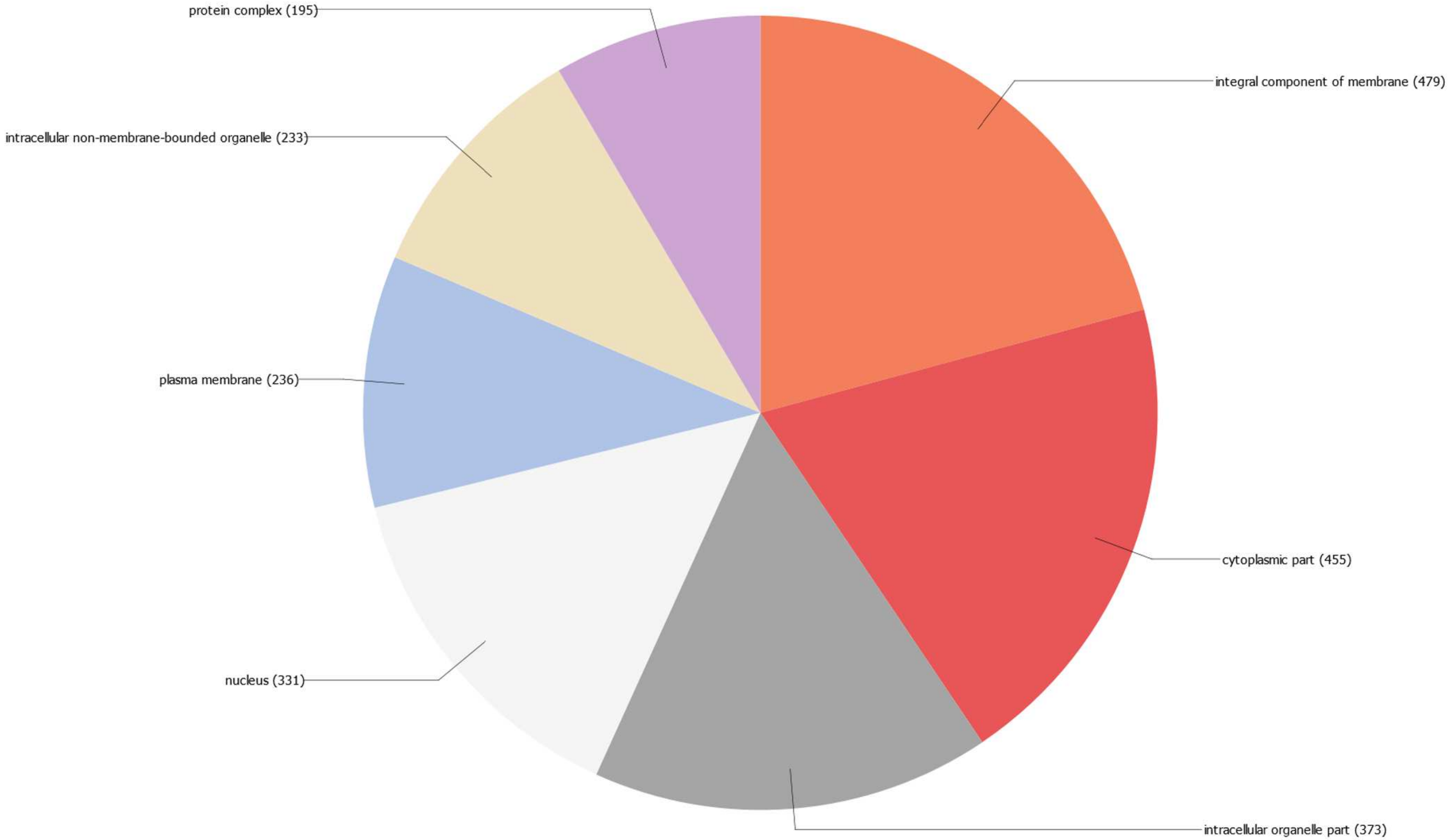
protein complex (195)
integral component of membrane (479)
intracellular non-membrane-bounded organelle (233)
plasma membrane (236)
cytoplasmic part (455)
nucleus (331)
intracellular organelle part (373)

Supplementary Figure S4. Results of the GO term annotation for targets of down-regulated miRNAs from P2 vs. P1 comparison. The GO terms annotation was performed by the Blast2GO software. Figure represents the pie chart graph of GO terms distribution from "Biological Process" (A), "Molecular Function" (B) and "Cellular Component" (C) category – numbers in brackets represent number of annotated carp sequences.

A)

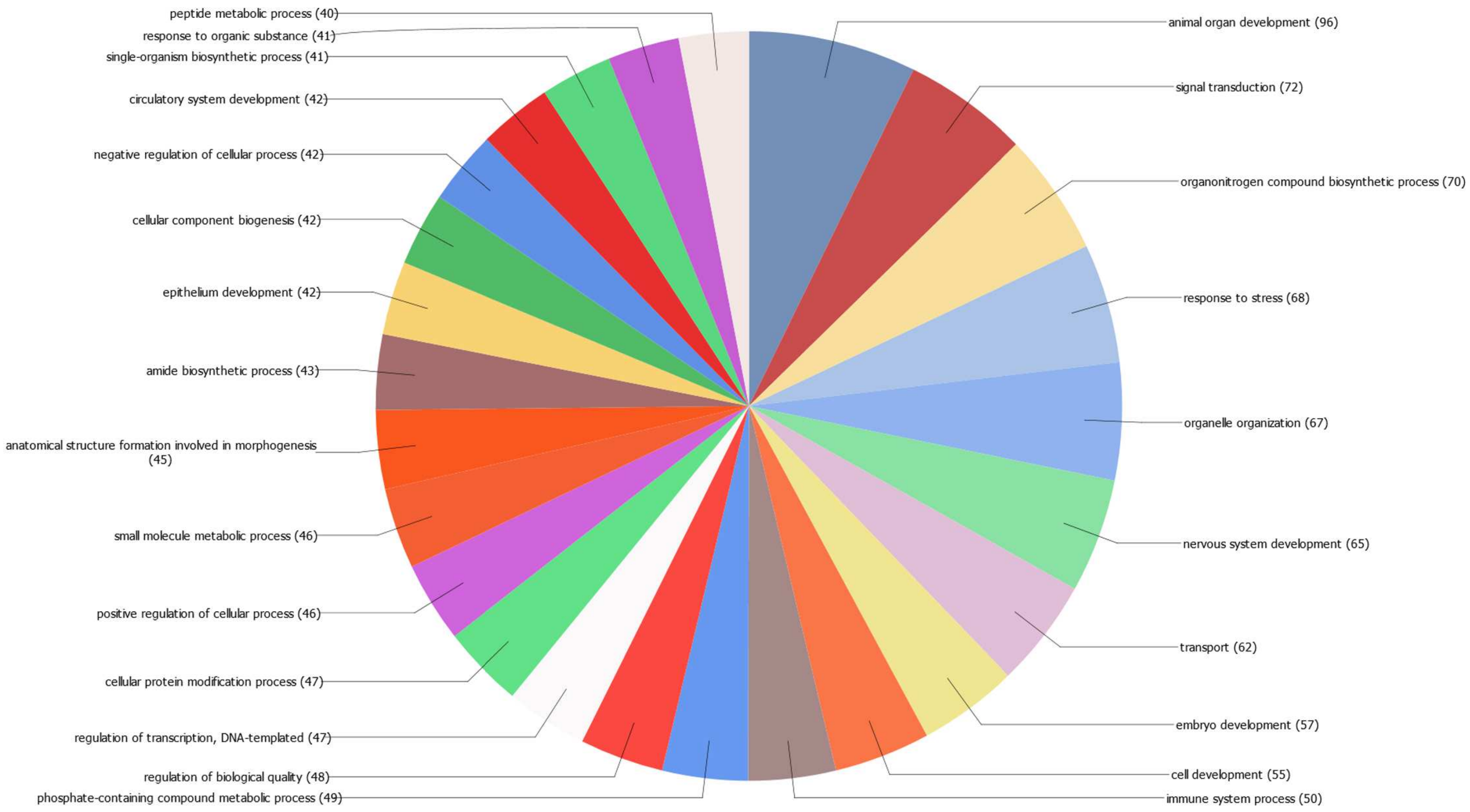

animal organ development (96)
signal transduction (72)
organonitrogen compound biosynthetic process (70)
response to stress (68)
organelle organization (67)
nervous system development (65)
transport (62)
embryo development (57)
cell development (55)
immune system process (50)
phosphate-containing compound metabolic process (49)
regulation of biological quality (48)
regulation of transcription, DNA-templated (47)
cellular protein modification process (47)
positive regulation of cellular process (46)
small molecule metabolic process (46)
anatomical structure formation involved in morphogenesis (45)
amide biosynthetic process (43)
epithelium development (42)
cellular component biogenesis (42)
negative regulation of cellular process (42)
circulatory system development (42)
single-organism biosynthetic process (41)
response to organic substance (41)
peptide metabolic process (40)

B)

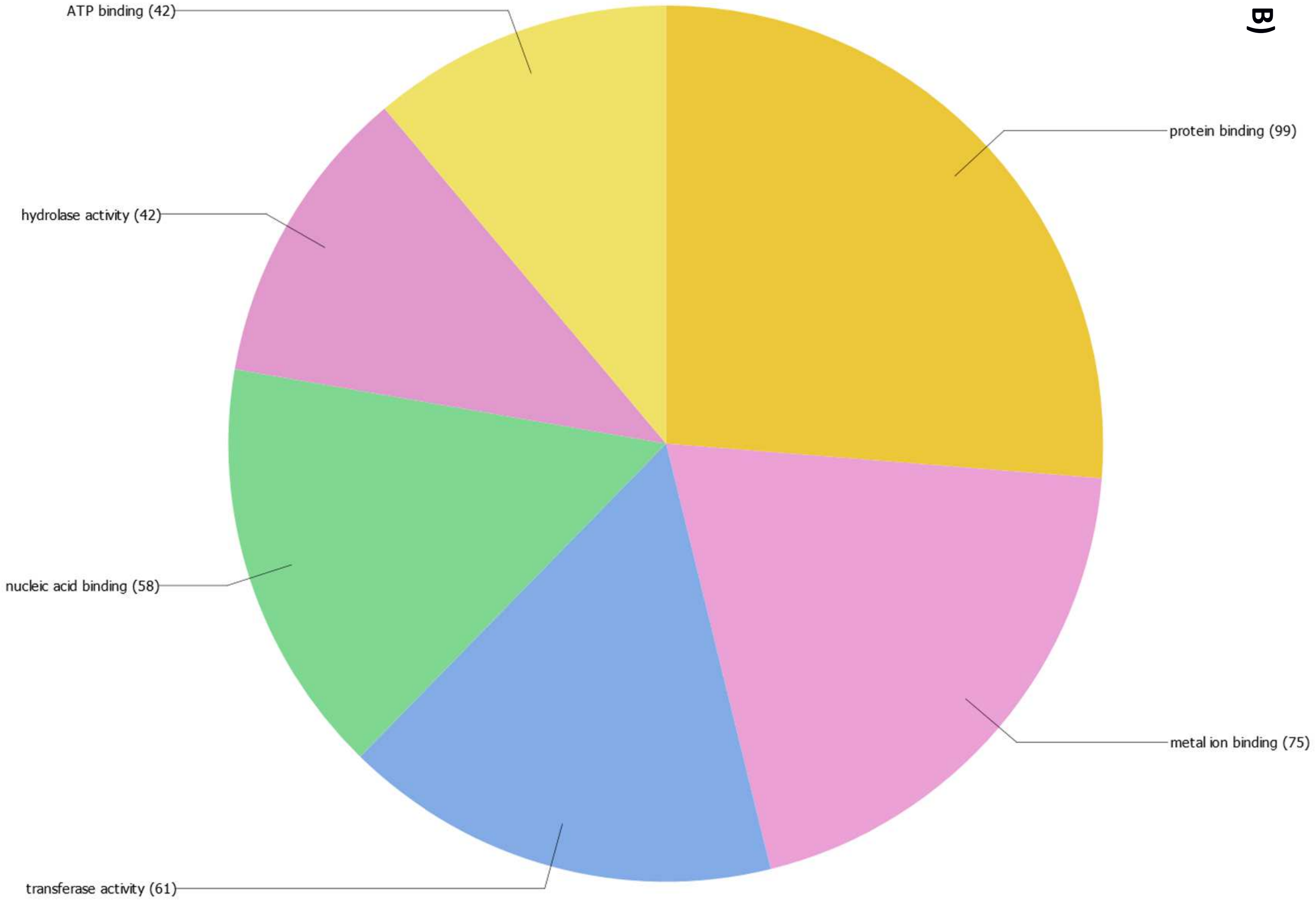
ATP binding (42)
protein binding (99)
hydrolase activity (42)
nucleic acid binding (58)
metal ion binding (75)
transferase activity (61)

C)

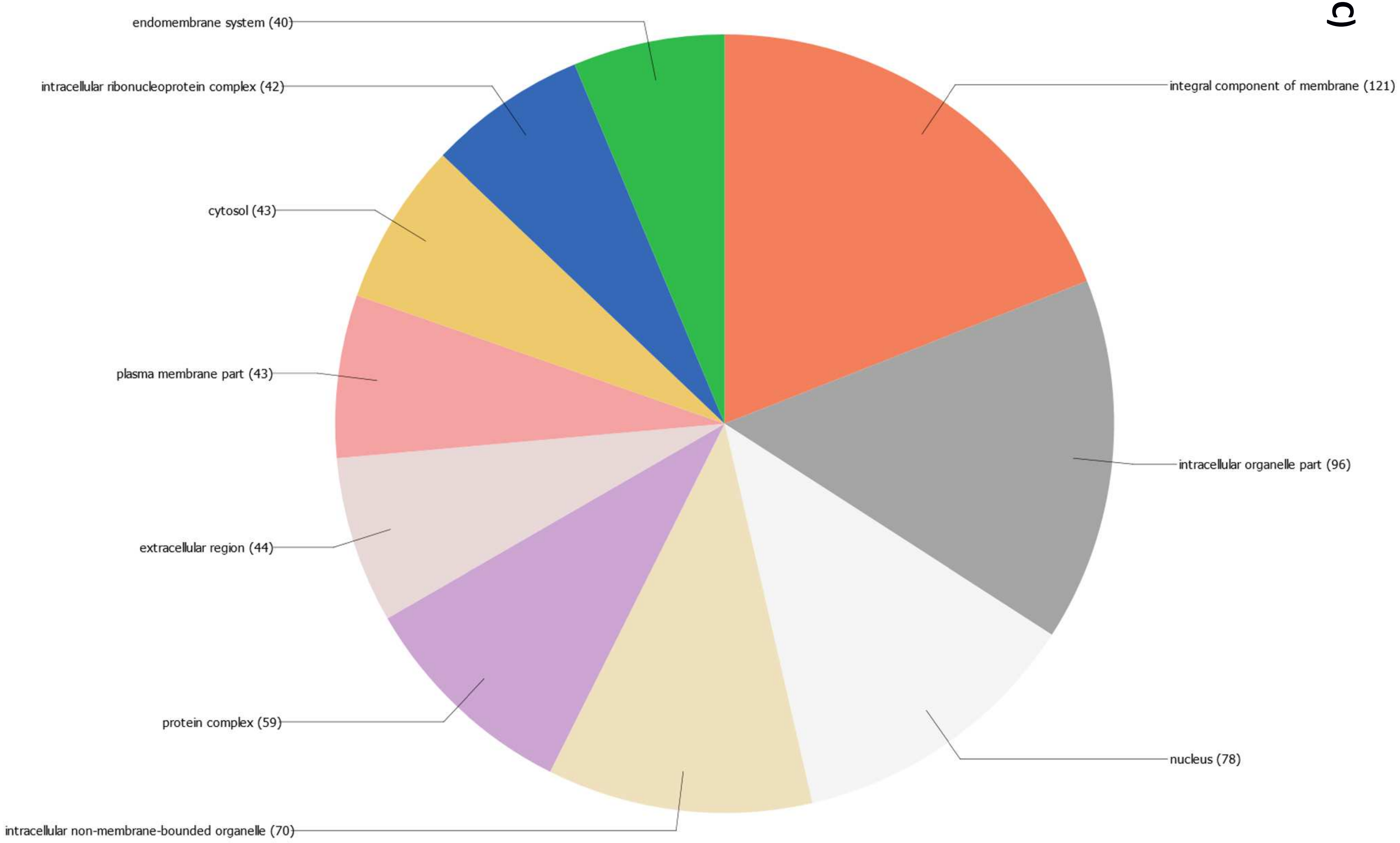
endomembrane system (40)
intracellular ribonucleoprotein complex (42)
cytosol (43)
plasma membrane part (43)
extracellular region (44)
protein complex (59)
intracellular non-membrane-bounded organelle (70)
nucleus (78)
intracellular organelle part (96)
integral component of membrane (121)

Supplementary Figure S4. Results of the GO term annotation for targets of up-regulated and down-regulated miRNAs from P3 vs. P1 comparison. The GO terms annotation was performed by the Blast2GO software. Figure represents the pie chart graph of GO terms distribution from "Biological Process" (A), "Molecular Function" (B) and "Cellular Component" (C) category – numbers in brackets represent number of annotated carp sequences.

A)

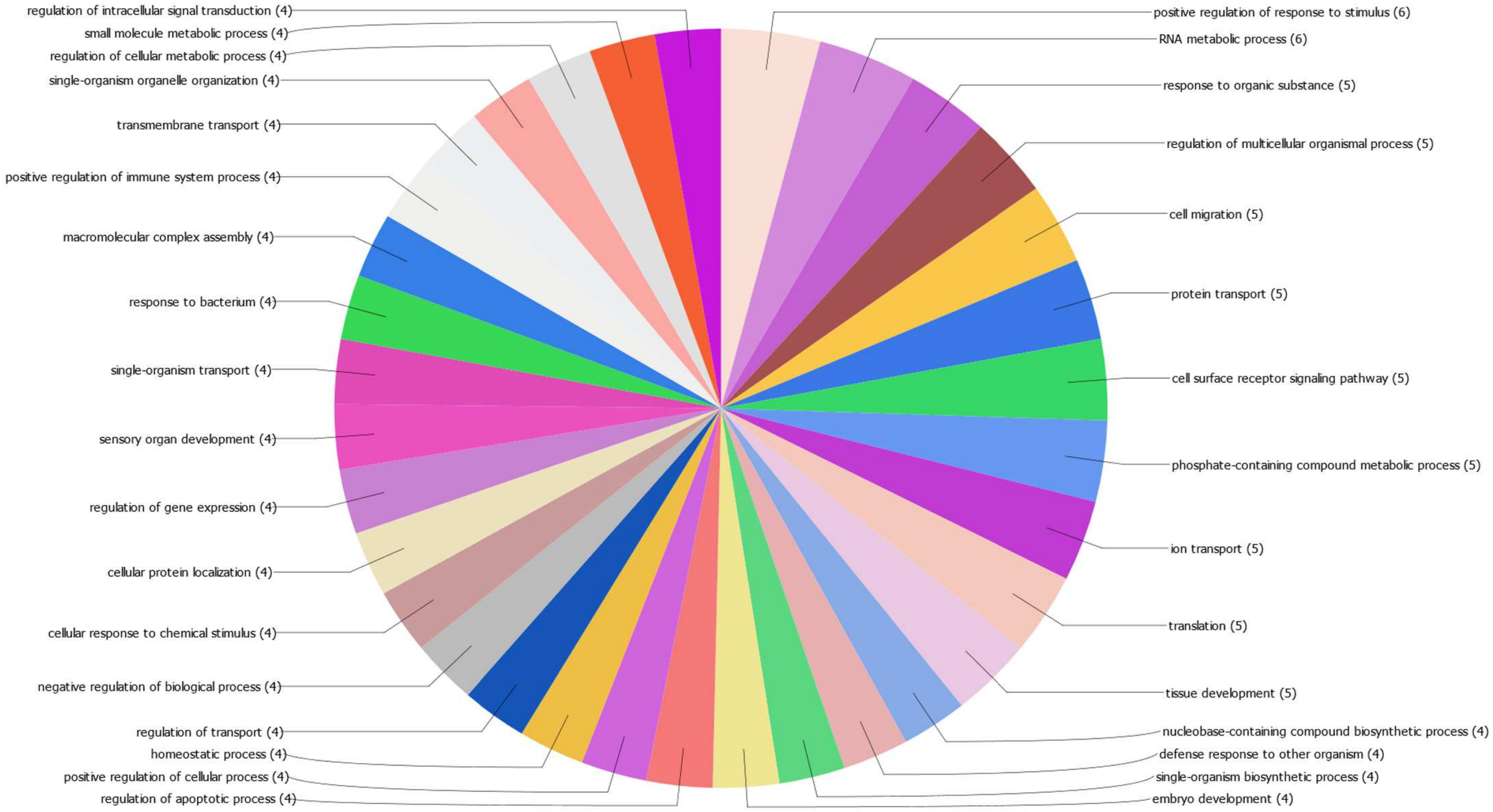

regulation of intracellular signal transduction (4)
small molecule metabolic process (4)
regulation of cellular metabolic process (4)
single-organism organelle organization (4)
transmembrane transport (4)
positive regulation of immune system process (4)
macromolecular complex assembly (4)
response to bacterium (4)
single-organism transport (4)
sensory organ development (4)
regulation of gene expression (4)
cellular protein localization (4)
cellular response to chemical stimulus (4)
negative regulation of biological process (4)
regulation of transport (4)
homeostatic process (4)
positive regulation of cellular process (4)
regulation of apoptotic process (4)
positive regulation of response to stimulus (6)
RNA metabolic process (6)
response to organic substance (5)
regulation of multicellular organismal process (5)
cell migration (5)
protein transport (5)
cell surface receptor signaling pathway (5)
phosphate-containing compound metabolic process (5)
ion transport (5)
translation (5)
tissue development (5)
nucleobase-containing compound biosynthetic process (4)
defense response to other organism (4)
single-organism biosynthetic process (4)
embryo development (4)

B)

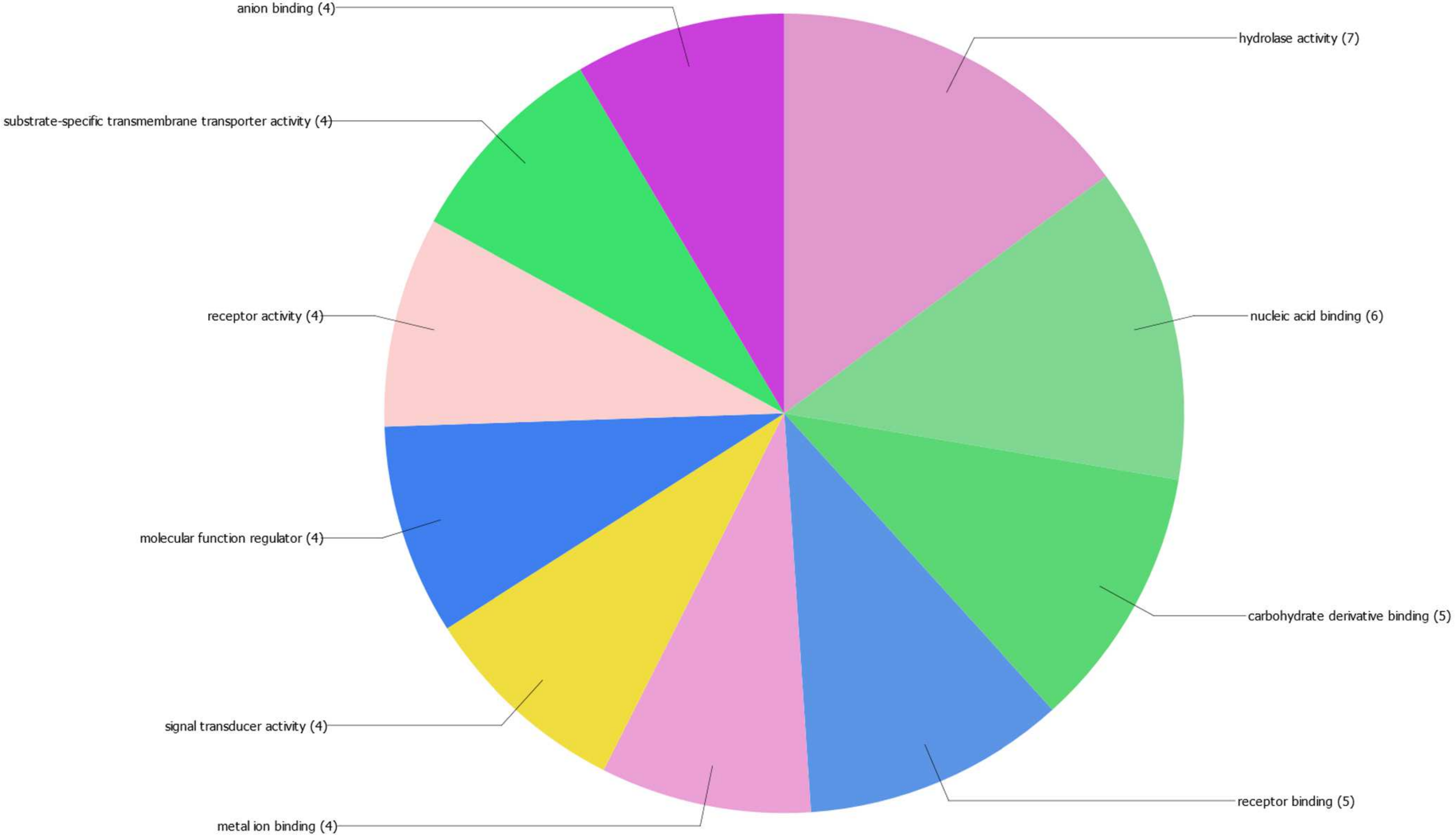

hydrolase activity (7)
nucleic acid binding (6)
carbohydrate derivative binding (5)
receptor binding (5)
metal ion binding (4)
signal transducer activity (4)
molecular function regulator (4)
receptor activity (4)
substrate-specific transmembrane transporter activity (4)
anion binding (4)

C)

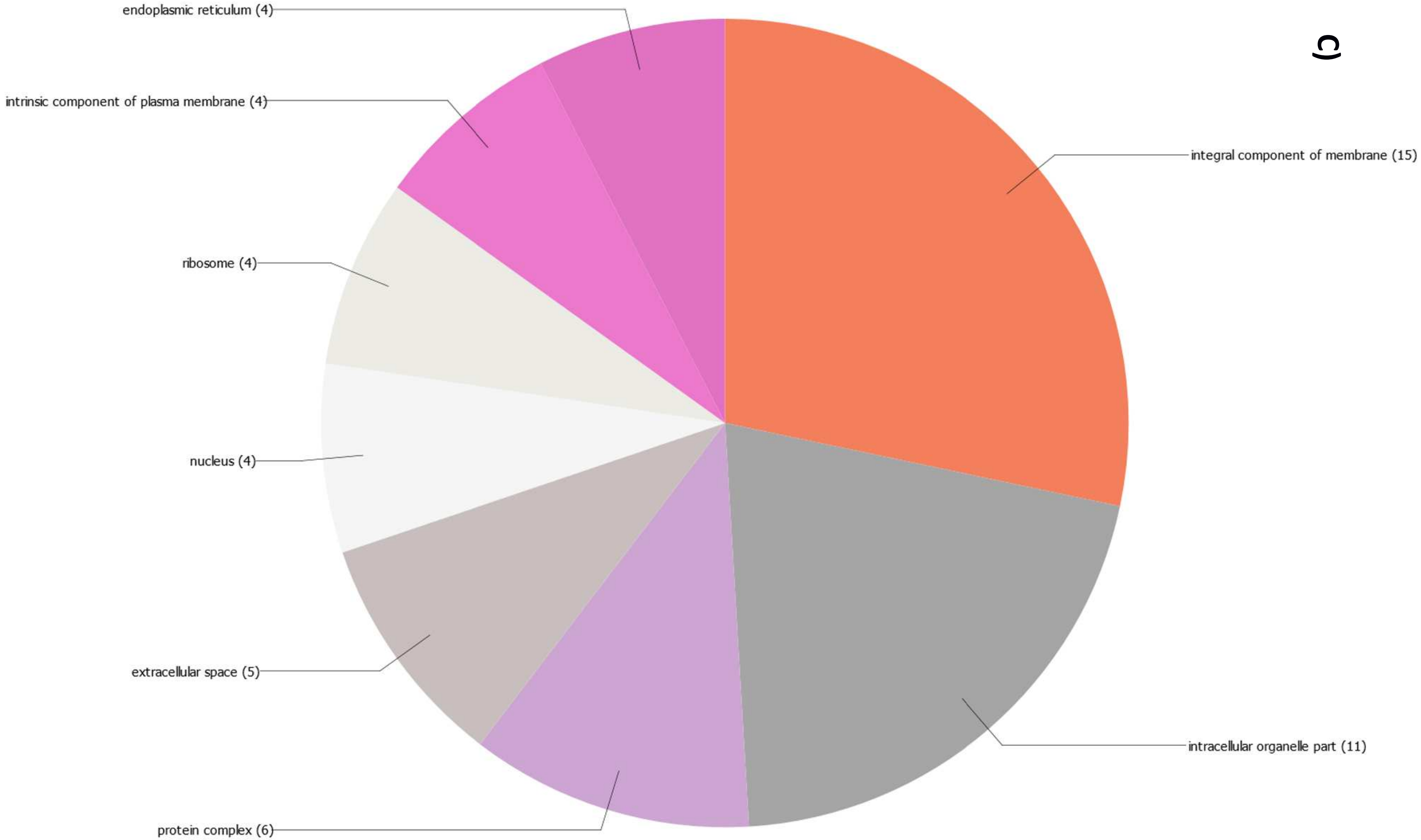
endoplasmic reticulum (4)
intrinsic component of plasma membrane (4)
ribosome (4)
nucleus (4)
extracellular space (5)
protein complex (6)
integral component of membrane (15)
intracellular organelle part (11)

Supplementary Figure S5. Results of GO term enrichment analysis for targets of carp up-regulated miRNAs (P2 vs. P1 phase comparison). The GO terms enrichment analysis was performed by the Blast2GO software. The targets predicted for carp up-regulated miRNAs were used as a test set. All carp protein coding sequences were used as background. Figure represent enriched GO terms from the "Biological Process", "Cellular Component" and "Molecular Function" categories. The size of dot denotes number of targets annotated by given GO term. The FDR ≤ 0.05 was set as the threshold for significant GO term enrichment.

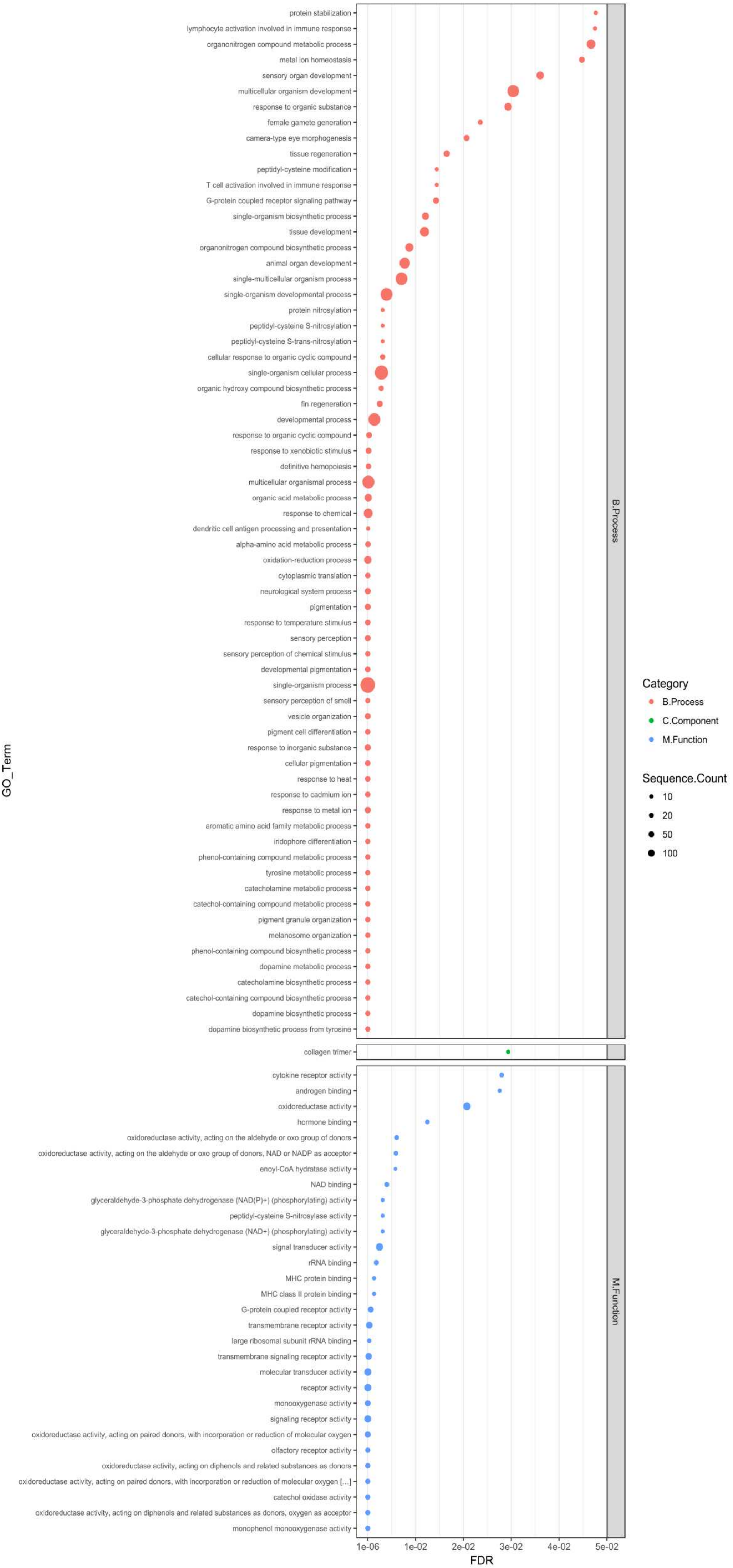

GO_Term
protein stabilization
lymphocyte activation involved in immune response
organonitrogen compound metabolic process
metal ion homeostasis
sensory organ development
multicellular organism development
response to organic substance
female gamete generation
camera-type eye morphogenesis
tissue regeneration
peptidyl-cysteine modification
T cell activation involved in immune response
G-protein coupled receptor signaling pathway
single-organism biosynthetic process
tissue development
organonitrogen compound biosynthetic process
animal organ development
single-multicellular organism process
single-organism developmental process
protein nitrosylation
peptidyl-cysteine S-nitrosylation
peptidyl-cysteine S-trans-nitrosylation
cellular response to organic cyclic compound
single-organism cellular process
organic hydroxy compound biosynthetic process
fin regeneration
developmental process
response to organic cyclic compound
response to xenobiotic stimulus
definitive hemopoiesis
multicellular organismal process
organic acid metabolic process
response to chemical
dendritic cell antigen processing and presentation
alpha-amino acid metabolic process
oxidation-reduction process
cytoplasmic translation
neurological system process
pigmentation
response to temperature stimulus
sensory perception
sensory perception of chemical stimulus
developmental pigmentation
single-organism process
sensory perception of smell
vesicle organization
pigment cell differentiation
response to inorganic substance
cellular pigmentation
response to heat
response to cadmium ion
response to metal ion
aromatic amino acid family metabolic process
iridophore differentiation
phenol-containing compound metabolic process
tyrosine metabolic process
catecholamine metabolic process
catechol-containing compound metabolic process
pigment granule organization
melanosome organization
phenol-containing compound biosynthetic process
dopamine metabolic process
catecholamine biosynthetic process
catechol-containing compound biosynthetic process
dopamine biosynthetic process
dopamine biosynthetic process from tyrosine
B.Process
collagen trimer
cytokine receptor activity
androgen binding
oxidoreductase activity
hormone binding
oxidoreductase activity, acting on the aldehyde or oxo group of donors
oxidoreductase activity, acting on the aldehyde or oxo group of donors, NAD or NADP as acceptor
enoyl-CoA hydratase activity
NAD binding
glyceraldehyde-3-phosphate dehydrogenase (NAD(P)+) (phosphorylating) activity
peptidyl-cysteine S-nitrosylase activity
glyceraldehyde-3-phosphate dehydrogenase (NAD+) (phosphorylating) activity
signal transducer activity
rRNA binding
MHC protein binding
MHC class II protein binding
G-protein coupled receptor activity
transmembrane receptor activity
large ribosomal subunit rRNA binding
transmembrane signaling receptor activity
molecular transducer activity
receptor activity
monooxygenase activity
signaling receptor activity
oxidoreductase activity, acting on paired donors, with incorporation or reduction of molecular oxygen
olfactory receptor activity
oxidoreductase activity, acting on diphenols and related substances as donors
oxidoreductase activity, acting on paired donors, with incorporation or reduction of molecular oxygen [...]
catechol oxidase activity
oxidoreductase activity, acting on diphenols and related substances as donors, oxygen as acceptor
monophenol monooxygenase activity
M.Function
1e-06
1e-02
2e-02
3e-02
4e-02
5e-02
FDR
Category
B.Process
C.Component
M.Function
Sequence.Count
10
20
50
100

Supplementary Figure S6. Results of GO term enrichment analysis for targets of carp down-regulated miRNAs (P2 vs. P1 phase comparison). The GO terms enrichment analysis was performed by the Blast2GO software. The targets predicted for carp down-regulated miRNAs were used as a test set. All carp protein coding sequences were used as background. Figure represent enriched GO terms from the “Biological Process”, “Cellular Component” and “Molecular Function” categories. The size of dot denotes number of targets annotated by given GO term. The FDR ≤ 0.05 was set as the threshold for significant GO term enrichment.

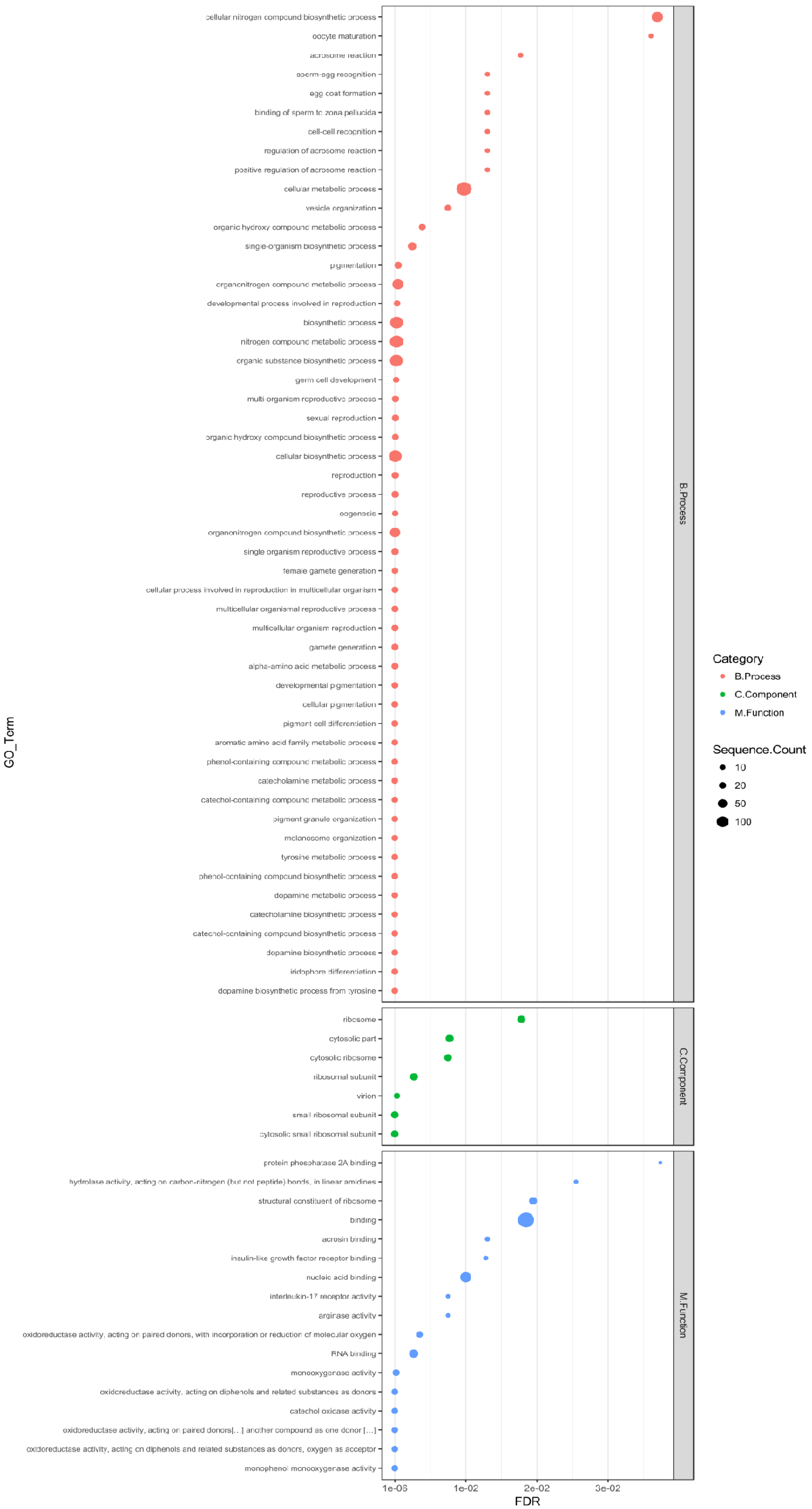
cellular nitrogen compound biosynthetic process
oocyte maturation
acrosome reaction
sperm-egg recognition
egg coat formation
binding of sperm to zona pellucida
cell-cell recognition
regulation of acrosome reaction
positive regulation of acrosome reaction
cellular metabolic process
vesicle organization
organic hydroxy compound metabolic process
single-organism biosynthetic process
pigmentation
organonitrogen compound metabolic process
developmental process involved in reproduction
biosynthetic process
nitrogen compound metabolic process
organic substance biosynthetic process
germ cell development
multi-organism reproductive process
sexual reproduction
organic hydroxy compound biosynthetic process
cellular biosynthetic process
reproduction
reproductive process
oogenesis
organonitrogen compound biosynthetic process
single organism reproductive process
female gamete generation
cellular process involved in reproduction in multicellular organism
multicellular organismal reproductive process
multicellular organism reproduction
gamete generation
alpha-amino acid metabolic process
developmental pigmentation
cellular pigmentation
pigment cell differentiation
aromatic amino acid family metabolic process
phenol-containing compound metabolic process
catecholamine metabolic process
catechol-containing compound metabolic process
pigment granule organization
melanosome organization
tyrosine metabolic process
phenol-containing compound biosynthetic process
dopamine metabolic process
catecholamine biosynthetic process
catechol-containing compound biosynthetic process
dopamine biosynthetic process
iridophore differentiation
dopamine biosynthetic process from tyrosine
B.Process
ribosome
cytosolic part
cytosolic ribosome
ribosomal subunit
virion
small ribosomal subunit
cytosolic small ribosomal subunit
C.Component
protein phosphatase 2A binding
hydrolase activity, acting on carbon-nitrogen (but not peptide) bonds, in linear amidines
structural constituent of ribosome
binding
acrosin binding
insulin-like growth factor receptor binding
nucleic acid binding
interleukin-17 receptor activity
arginase activity
oxidoreductase activity, acting on paired donors, with incorporation or reduction of molecular oxygen
RNA binding
monooxygenase activity
oxidoreductase activity, acting on diphenols and related substances as donors
catechol oxidase activity
oxidoreductase activity, acting on paired donors[...] another compound as one donor [...]
oxidoreductase activity, acting on diphenols and related substances as donors, oxygen as acceptor
monophenol monooxygenase activity
M.Function
GO_Term
1e-06
1e-02
2e-02
3e-02
FDR
Category
B.Process
C.Component
M.Function
Sequence.Count
10
20
50
100

Supplementary Figure S7. The enriched KEGG pathway obtained for targets of down-regulated carp miRNAs (P2 vs. P1 phase comparison) – "Necroptosis" (dre04217). The KEGG pathway enrichment analysis was performed with the clusterProfiler R package. The zebrafish KEGG pathways served as reference. The targets predicted for down-regulated miRNAs which mapped on given pathway are marked as pink boxes.

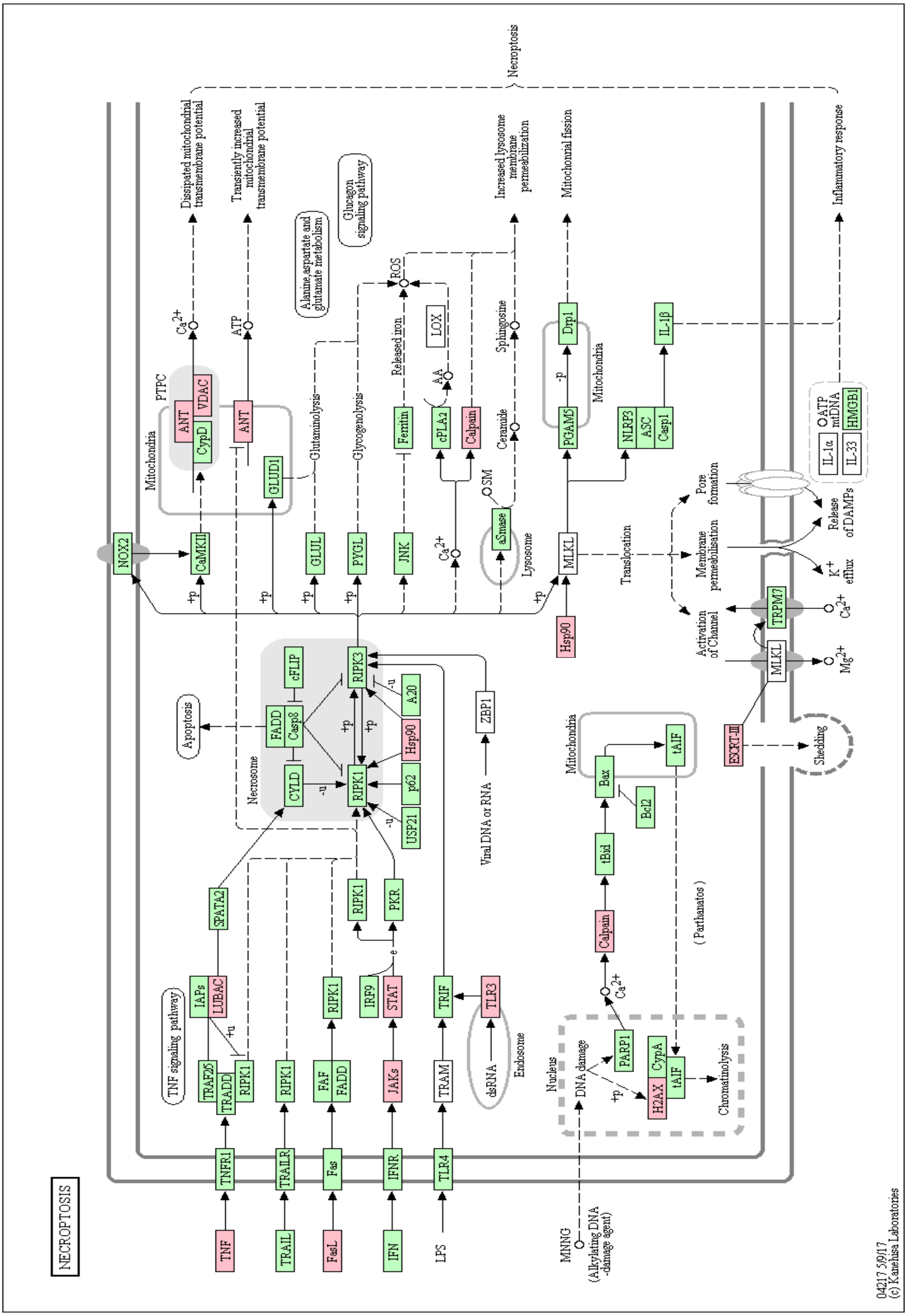

Supplementary Figure S8. The enriched KEGG pathway obtained for targets of up-regulated carp miRNAs (P2 vs. P1 phase comparison) – "Fatty acid metabolism" (dre01212). The KEGG pathway enrichment analysis was performed with the clusterProfiler R package. The zebrafish KEGG pathways served as reference. The targets predicted for up-regulated miRNAs which mapped on given pathway are marked as pink arrows.

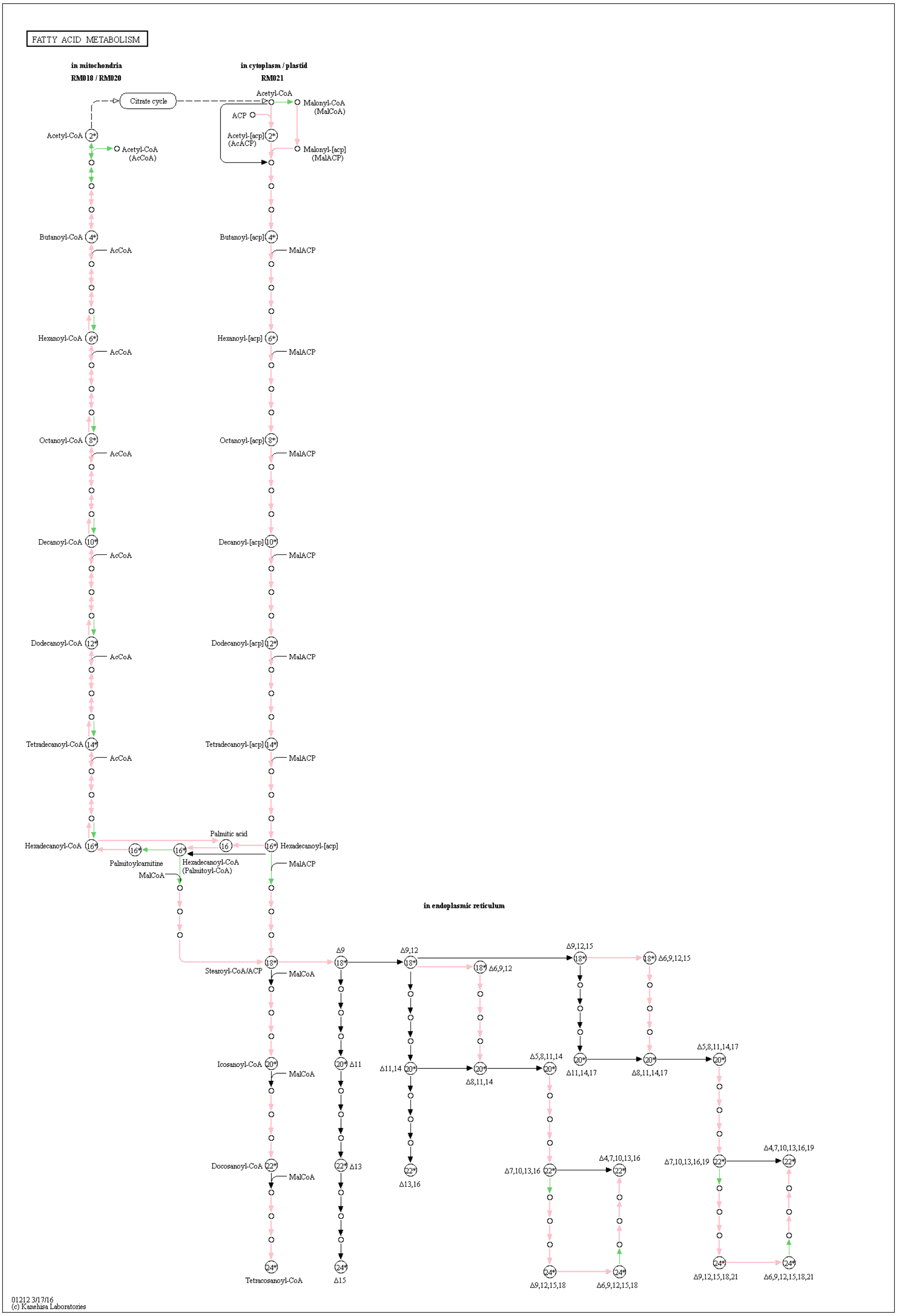

Supplementary Figure S9. Predicted hairpin structures of novel carp miRNA molecules identified in samples from phase P1. Dominant form of the mature miRNAs is indicated in blue. Opposite strand miRNAs ("miRNA*") are indicated in red. The novel precursors were visualized using the UEA sRNA Workbench method.

A)

Novel-miRNA1

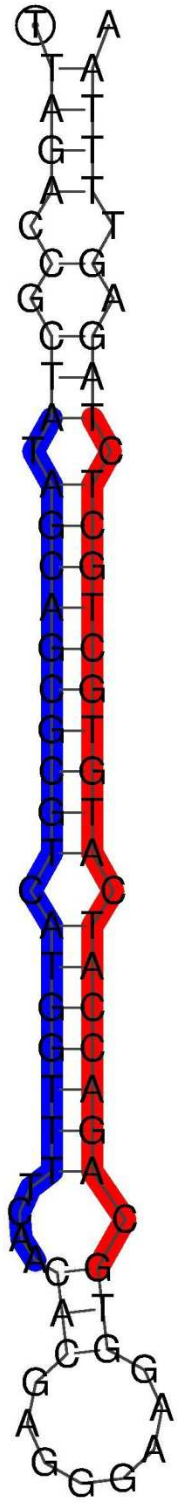

B)

Novel-miRNA2

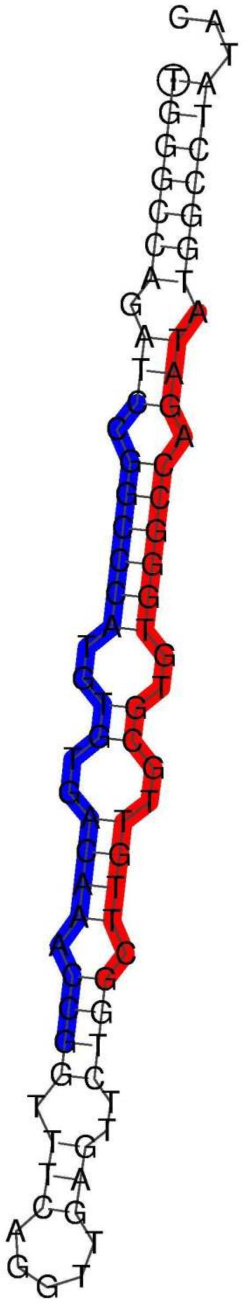

C)

Novel-miRNA3

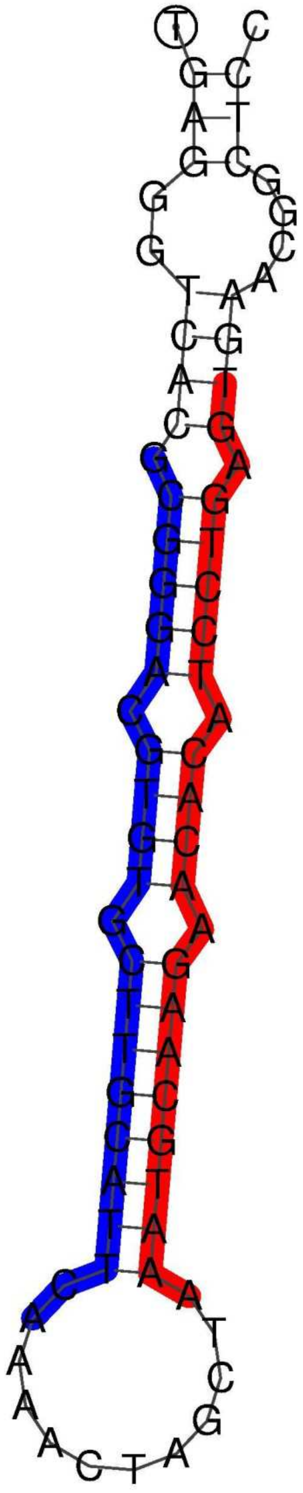

D)

Novel-miRNA4

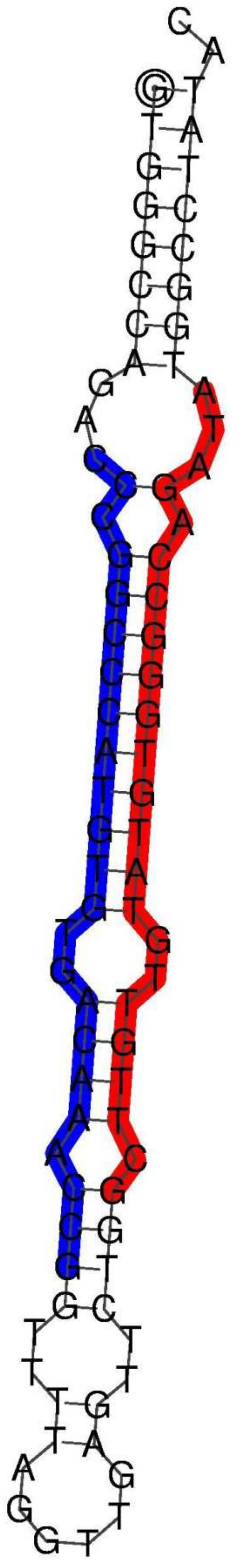

E)

Novel-miRNA5

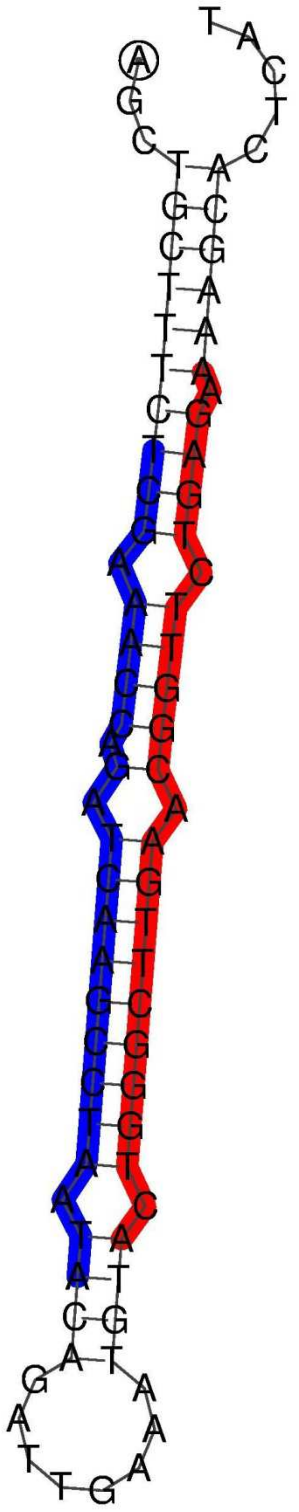

F)

Novel-miRNA6

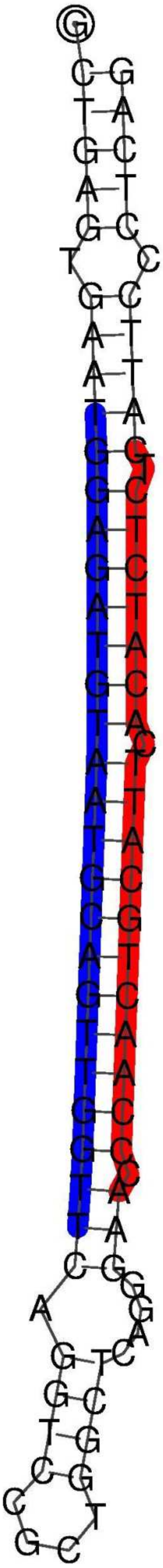

G)

Novel-miRNA7

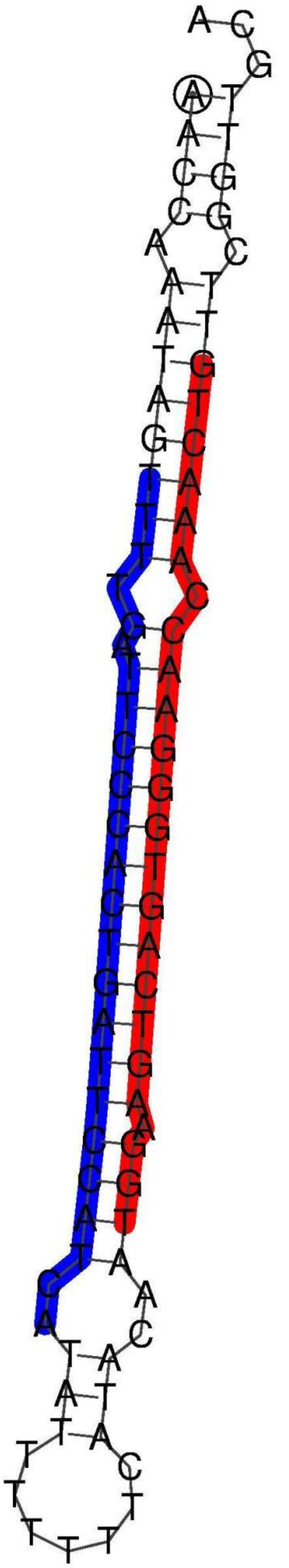

H)

Novel-miRNA8

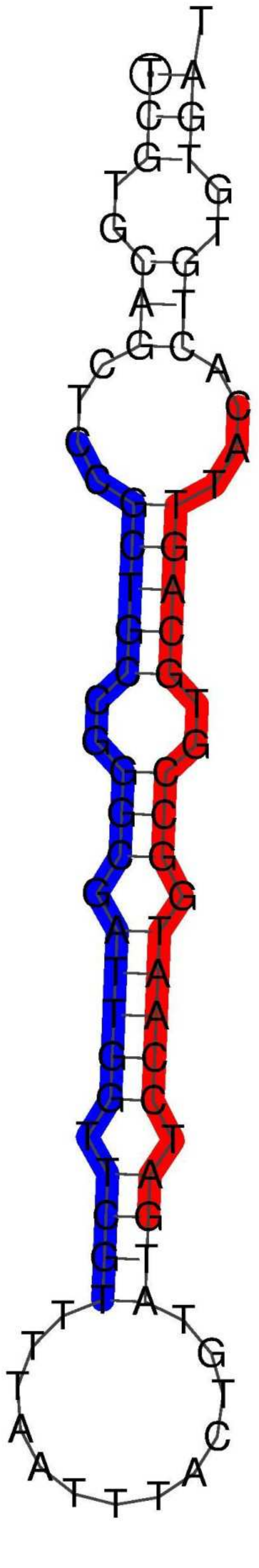

Supplementary Figure S10. Predicted hairpin structures of novel carp miRNA molecules identified in samples from phase P2. Dominant form of the mature miRNAs is indicated in blue. Opposite strand miRNAs ("miRNA*") are indicated in red. The novel precursors were visualized using the UEA sRNA Workbench method.

A)

Novel-miRNA1

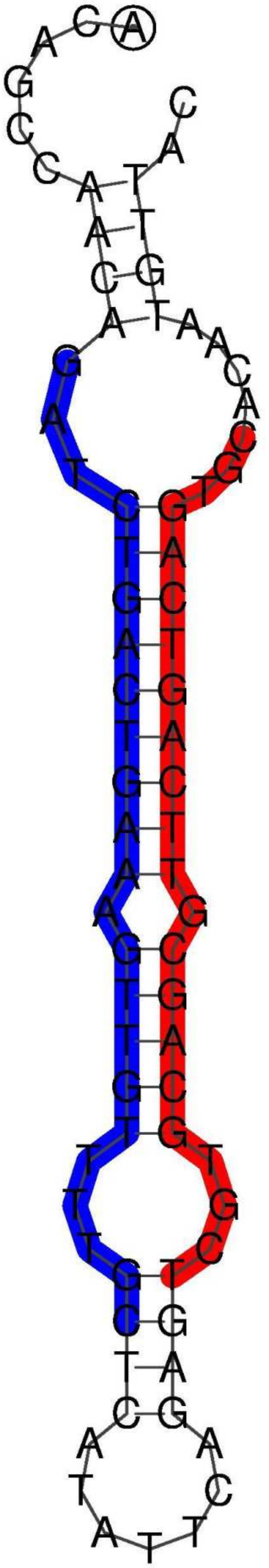

B)

Novel-miRNA2

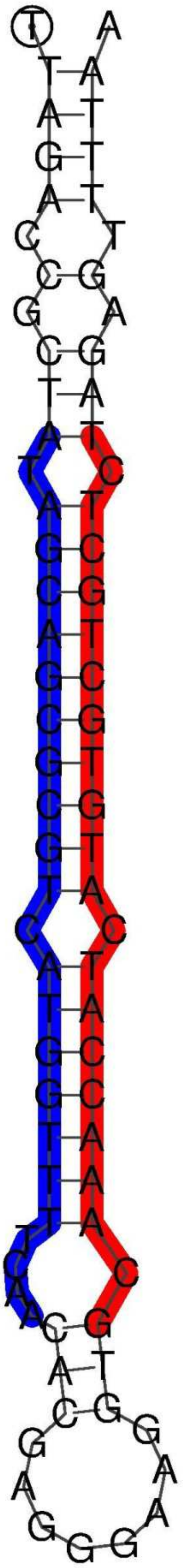

C)

Novel-miRNA3

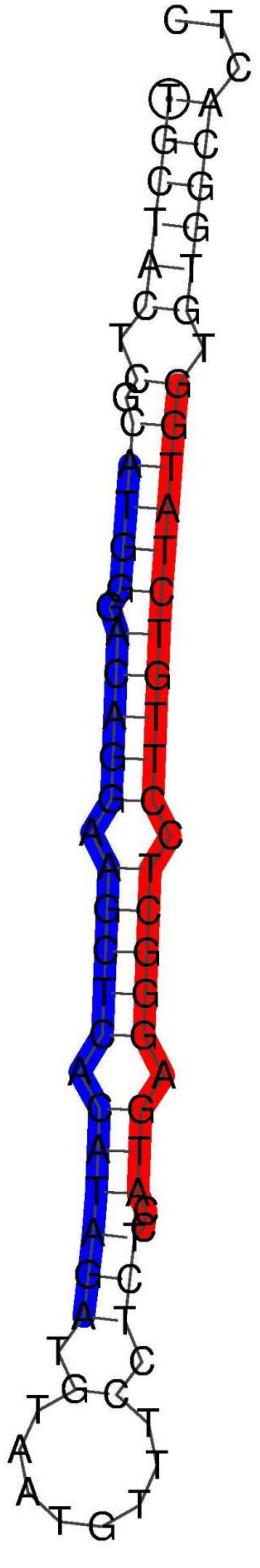

D)

Novel-miRNA4

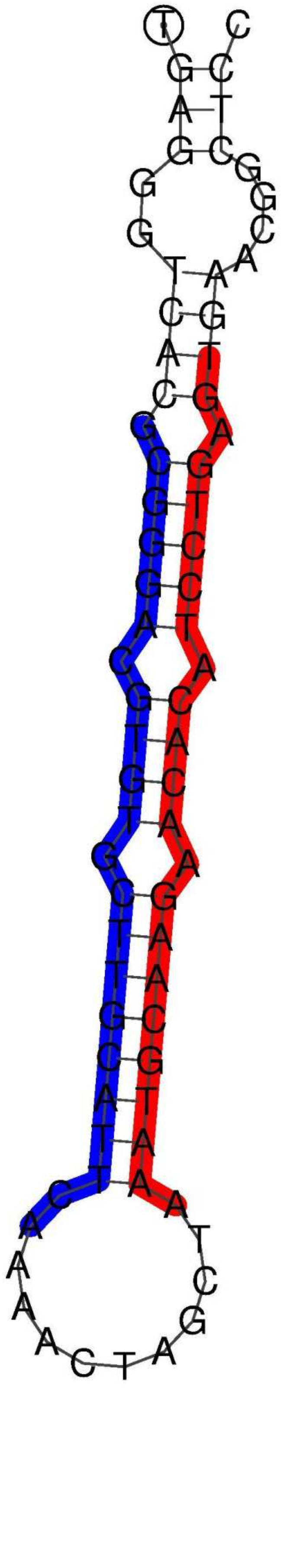

E)

Novel-miRNA5

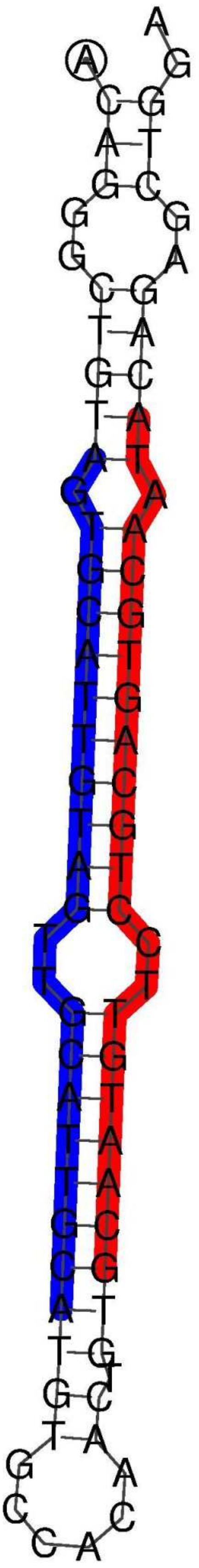

F)

Novel-miRNA6

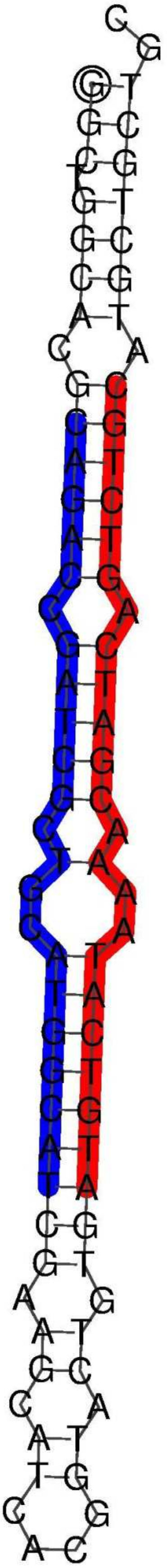

G)

Novel-miRNA7

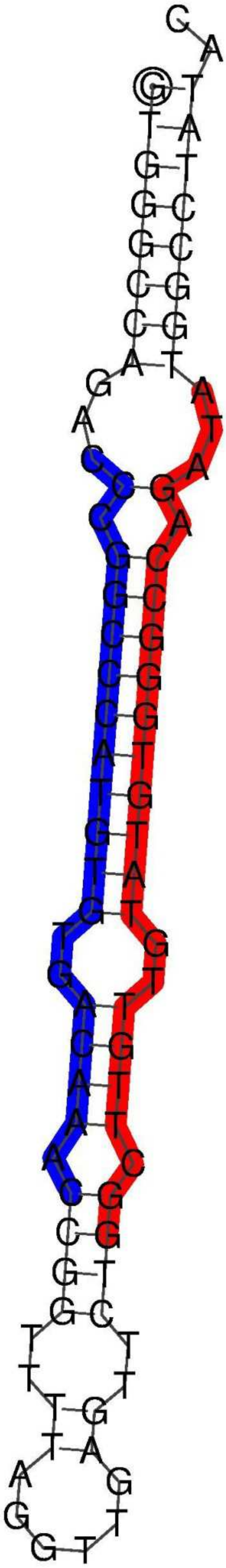

H)

Novel-miRNA8

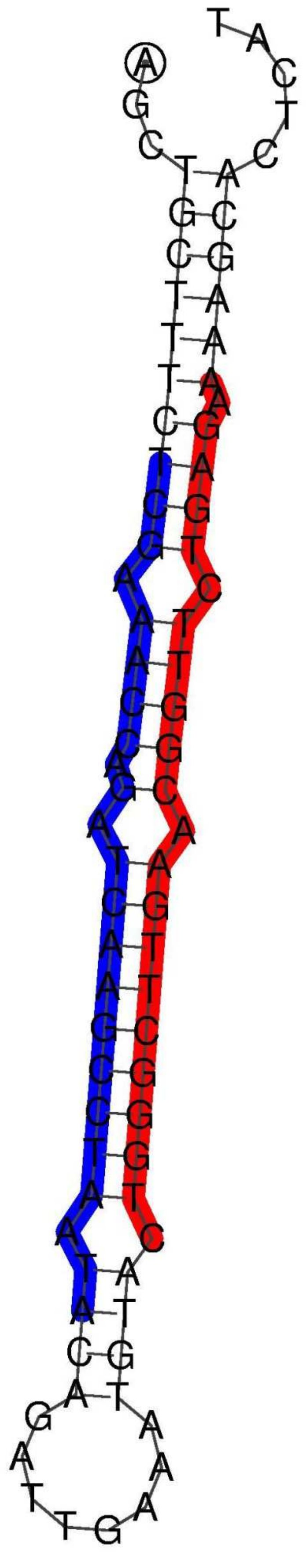

I)

Novel-miRNA9

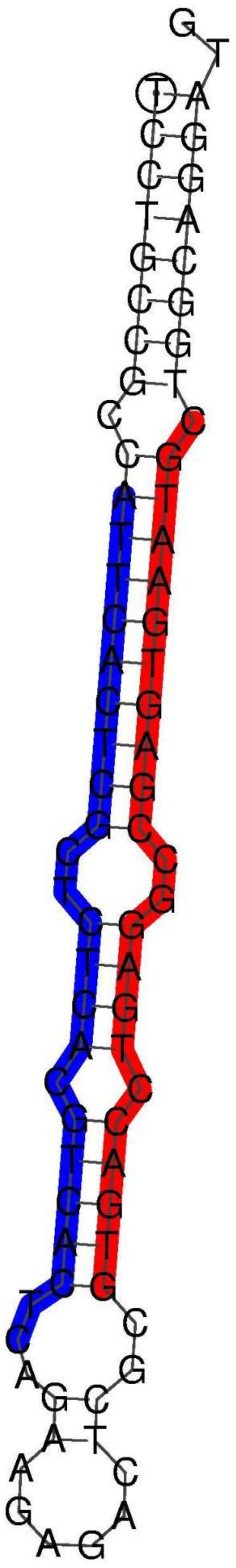

J)

Novel-miRNA10

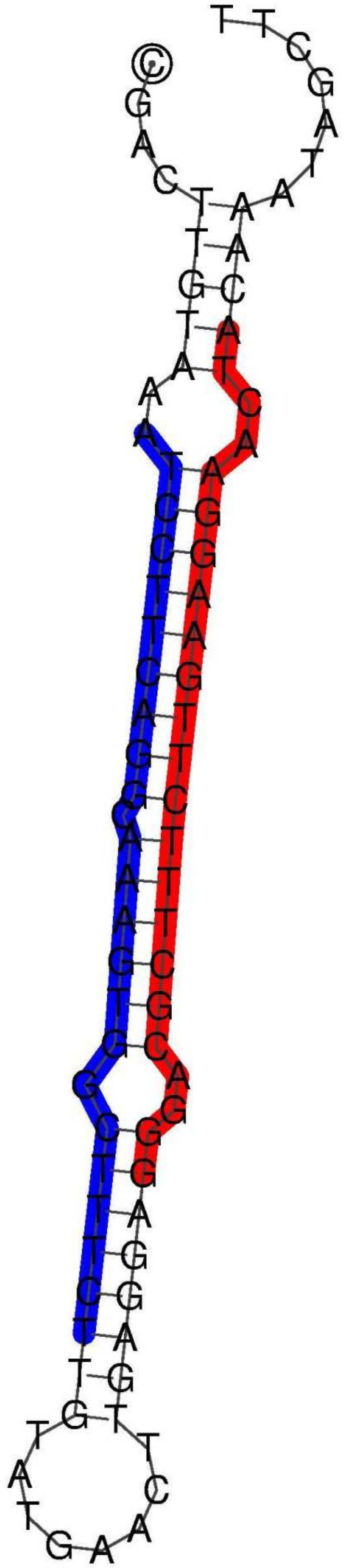

Supplementary Figure S11. Predicted hairpin structures of novel carp miRNA molecules identified in samples from phase P3. Dominant form of the mature miRNAs is indicated in blue. Opposite strand miRNAs ("miRNA*") are indicated in red. The novel precursors were visualized using the UEA sRNA Workbench method.

A)

Novel-miRNA1

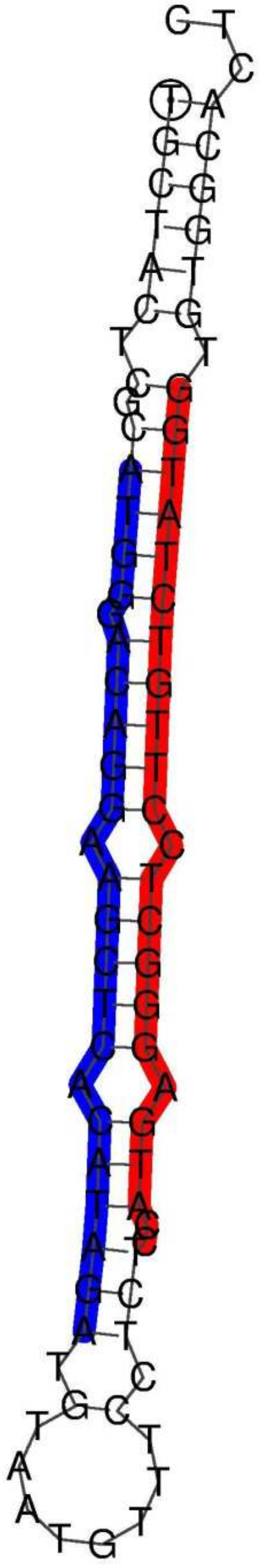

B)

Novel-miRNA2

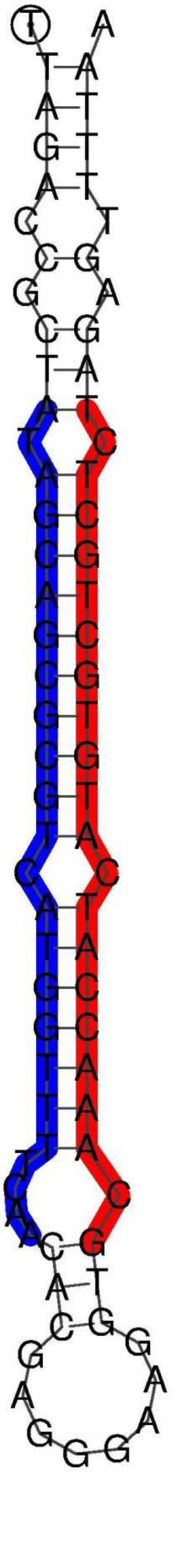

C)

Novel-miRNA3

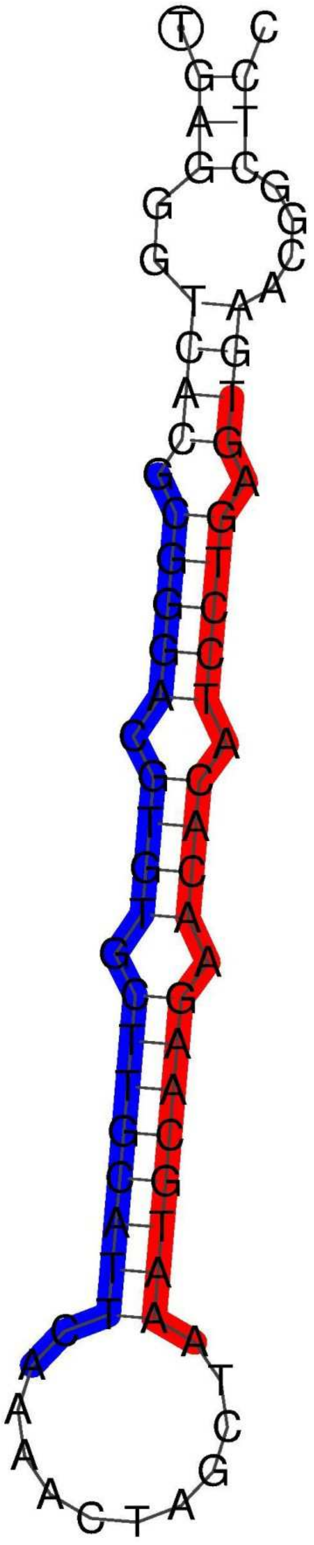

D)

Novel-miRNA4

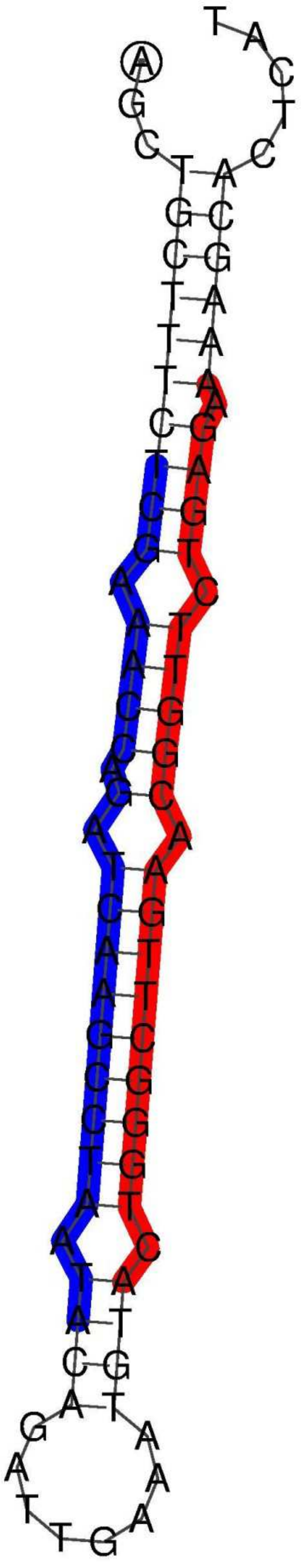

E)

Novel-miRNA5

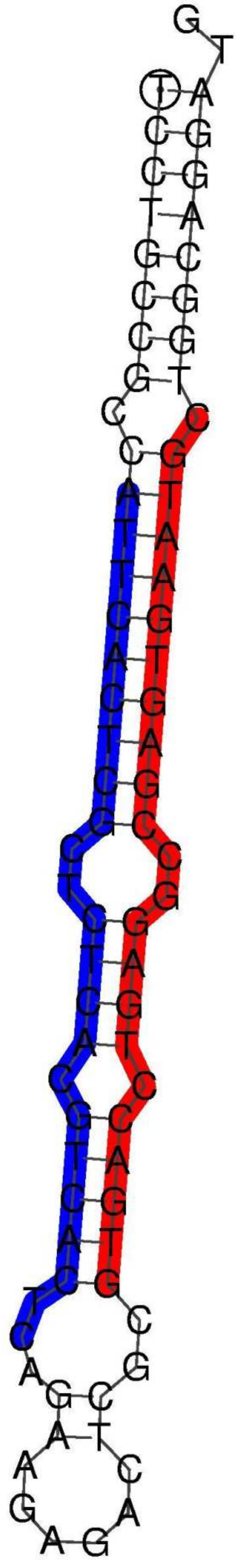

Supplement: Supplementary file 1 — Figure S1-S11. All supplementary figures. Table S1. Common carp and D. rerio reference sequences used for sRNA data processing - BlastN search. (PDF 4341 kb) [file 12864_2018_5266_MOESM1_ESM.pdf]
